# Regulating transcriptional activity by phosphorylation: A new mechanism for the ARX homeodomain transcription factor

**DOI:** 10.1371/journal.pone.0206914

**Published:** 2018-11-12

**Authors:** Tessa Mattiske, May H. Tan, Oliver Dearsley, Desiree Cloosterman, Charles S. Hii, Jozef Gécz, Cheryl Shoubridge

**Affiliations:** 1 Adelaide Medical School, University of Adelaide, Adelaide, Australia; 2 Robinson Research Institute, University of Adelaide, Adelaide, Australia; 3 Department of Immunopathology, SA-Pathology, Adelaide, Australia; 4 Healthy Mothers and Babies, South Australian Health and Medical Research Institute, Adelaide, Australia; Stanford University School of Medicine, UNITED STATES

## Abstract

*Aristaless*-related homeobox (*ARX*) gene encodes a paired-type homeodomain transcription factor with critical roles in development. Here we identify that ARX protein is phosphorylated. Using mass spectrometry and *in vitro* kinase assays we identify phosphorylation at serines 37, 67 and 174. Through yeast-2-hybrid and CoIP we identified PICK1 (Protein interacting with C kinase 1) binding with the C-terminal region of ARX. PICK1 is a scaffold protein known to facilitate phosphorylation of protein partners by protein kinase C alpha (PRKCA). We confirm that ARX is phosphorylated by PRKCA and demonstrate phosphorylation at serine 174. We demonstrate that phosphorylation is required for correct transcriptional activity of the ARX protein using transcriptome-wide analysis of gene expression of phospho-null mutants (alanines replacing serines) compared to ARX wild-type (ARX-WT) overexpressed in pancreatic alpha TC cells. Compared to untransfected cells, ARX-WT overexpression significantly altered expression of 70 genes (Log2FC >+/-1.0, P-value <0.05). There were fewer genes with significantly altered expression compared to untransfected cells with the double phospho-null mutant Ser37Ala+Ser67Ala (26%) and Ser174Ala (39%), respectively. We demonstrate that the c-terminal region of ARX required to bind PICK1 causes a shift in PICK1 subcellular localisation to the nucleus to co-locate with the ARX protein, and truncation of this C-terminal region leads to the same loss of transcriptional activation as S174A mutant. In conclusion, we show that ARX is phosphorylated at several sites and that this modification affects its transcriptional activity.

## Introduction

The *Aristaless*-related homeobox (*ARX*) gene [NM_139058.2] is a member of the paired-type homeodomain transcription factor family, with critical roles on development. *ARX* expression is restricted predominately to the brain (highest in the fetal brain), with low levels of expression in the pancreas, testes, skeletal muscle and highly expressed in ovaries. Mutations in this gene (MIM 30082) are a heritable cause of X-linked intellectual disability with or without other comorbidities including epilepsy, infantile spasms, dystonia, dysarthria, autism or lissencephaly [1–4]. The most striking consequences of loss of ARX function impact the development of the brain, testis in both mouse and human [2, 5, 6]. However, ARX also plays a critical role in the developmental determination and maintenance of alpha cell fates in the pancreas [7]. Arx-deficient mice develop early-onset hypoglycaemia, dehydration and weakness, and die 2 days after birth likely due to the lack of Arx function in the pancreas leading to a near-complete deficiency of alpha cells and a concomitant increase in beta cells [7].

Transcriptional activity of the ARX protein is largely defined by the presence of a conserved paired-type homeodomain, involved in binding to specific DNA sequences to regulate the expression of subsets of target genes. The molecular mechanism that homeodomain proteins, such as ARX, employ to regulate transcription of specific target genes is still poorly understood. Other components of the protein may contribute to the transcriptional regulation capacity. For example, the ARX protein also contains an *Aristaless* domain, which is present in over half of the members of the paired class homeodomain family [8]. This domain is located at the carboxyl end of ARX protein. The function of this domain is not well understood, although a number of studies have indicated a role in transcription activation [7, 9–12]. Four hydrophobic polyalanine tracts located throughout the ARX protein are suggested to be involved in protein-protein and protein-DNA interactions and stabilise the interaction between transcription regulators and/or DNA [13]. Located near the N-terminus of ARX is a highly conserved octapeptide domain. This sequence shares high similarity with the Engrailed homology repressor domain (eh1) known to be involved in transcriptional repression both *in vitro* and *in vivo*. This domain recruits Grouch/transducing-like enhancer of split (TLE) co-factor protein (TLE1-4), which modulates transcription repression activity [12].

Similar to many transcription factors, there is evidence to support that ARX transcriptional function might be regulated through post-translational modification. A study examining the potential role of CDKL5 mutants in phosphorylating MECP2 or ARX has indicated that although ARX does undergo *in vivo* phosphorylation when overexpressed in HEK293T cells, CDKL5 was not the kinase responsible [14]. The kinase phosphorylating ARX is unknown, as is the effect of this modification on its function. In this study, we have established that ARX is phosphorylated at multiple sites, including serine 37, 67 and 174. We have identified protein interacting with C kinase 1 (PICK1) as a novel protein partner of ARX using yeast-2 hybrid and co-immunoprecipitation studies. PICK1 is a scaffolding protein first identified as an interaction partner for protein kinase C alpha (PRKCA) [15]. PICK1 is known to facilitate the phosphorylation of its substrate by PRKCA [16]. The PKC family consists of ubiquitously expressed Ca^2+^-dependent/independent, phospholipid-dependent serine/threonine kinases that play key roles in a large number of biological processes, including cell growth and differentiation. Our study has demonstrated that ARX is a novel substrate of PRKCA, with phosphorylation at serine 174. We demonstrate that ARX is phosphorylated at several sites and that this modification is important for aspects of transcription activity of ARX.

## Results

### Multiple phosphorylation sites exist in ARX

**Fig 1:**
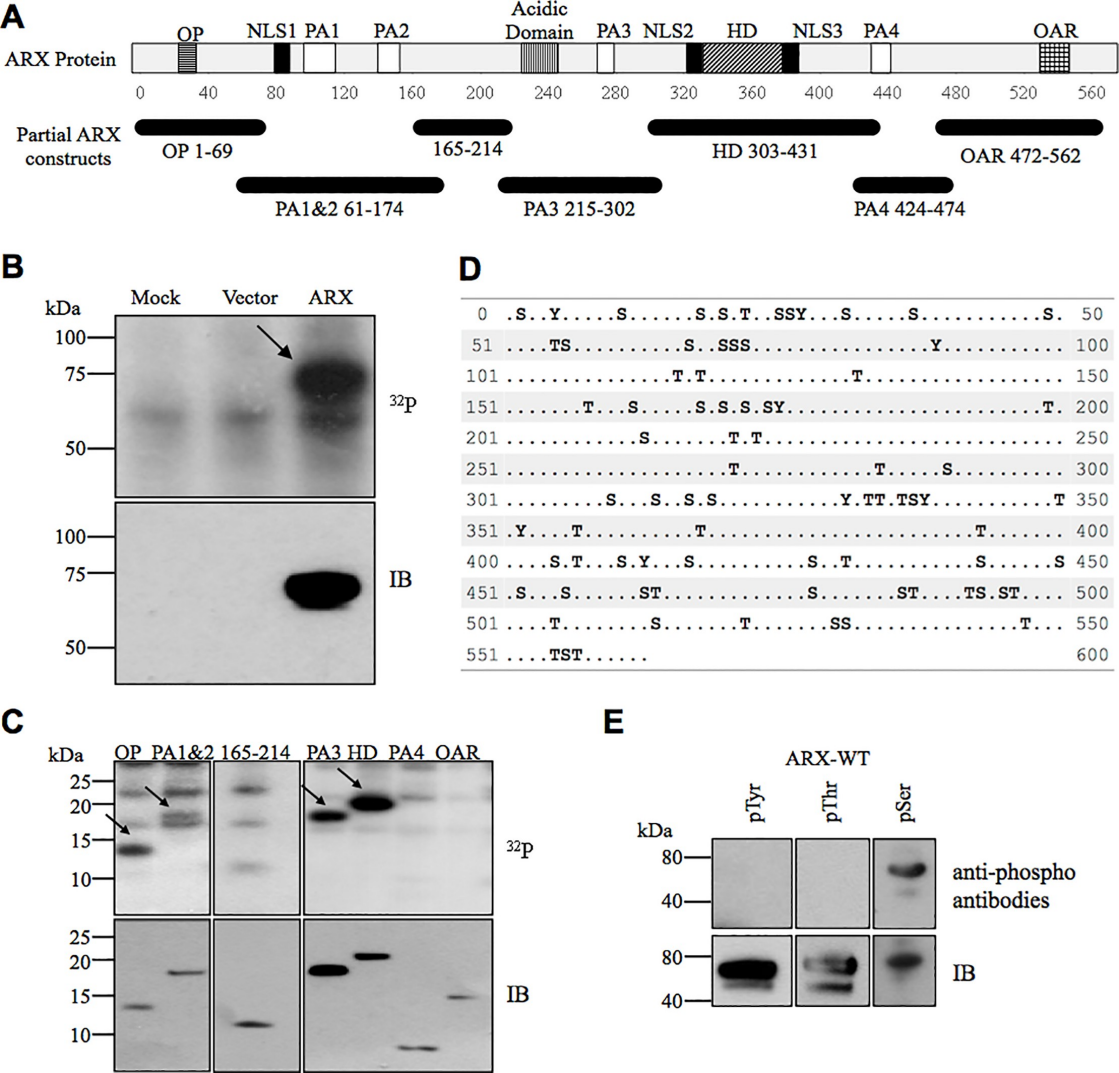
Multiple phosphorylation sites exist in ARX. (A) Schematic diagram of the ARX protein structure and position of partial constructs. Known functional domains are highlighted, octapeptide (OP) in horizontal stripes, nuclear localisation sequences (NLS) in black, polyalanine tracts (PA) in white, acidic domain in vertical stripes, homeodomain (HD) in diagonal stripes and Aristaless domain (OAR) in crosshatching. Partial constructs are named after the functional domain or the amino acids included and are shown below the protein. (B) HEK293T cells transfected with Myc-tagged full-length ARX construct were [^32^P] labelled, lysed and immunoprecipitated with an anti-Myc antibody and subjected to SDS-PAGE. Phosphorylated proteins visualised by autoradiography (arrow in upper panel). Presence of Myc-ARX protein was confirmed by immunoblotting (IB) using an anti-Myc HRP conjugated antibody (lower panel). Cells transfected with pCMV-Myc expression vector and untransfected cells were included as controls. These results are representative of at least two independent experiments. (C) Partial ARX constructs subjected to *in vivo* labelling assay as described for (B). (D) Serine, threonine, and tyrosine amino acids highlighted within the ARX protein. E) Immunoprecipitated Myc-ARX proteins were separated by SDS-PAGE and transferred to nitrocellulose membrane for immunoblotting with a 4G10 anti-phosphoTyrosine antibody (pTyr), an anti-phosphoThreonine antibody (pThr) or an anti-phosphoserine antibody (pSer). Presence of ARX was confirmed by immunoblotting using an anti-Myc antibody (bottom panel). Treated HEK 293T cells were included as positive controls; Pervanadate for pTry and CalyculinA for pThr (S1 Fig). Separate blots are boxed with a black outline.

In the presence of radiolabelled ([^32^P]) orthophosphoric acid, immunoprecipitated recombinant Myc-tagged full-length ARX protein overexpressed in HEK293T cells shows a positive signal of phosphorylation (Fig 1B, top panel). No phosphorylation signal was detected in Myc-vector transfected cells or mock-transfected cells. To narrow down the possible phosphorylation site(s) in ARX we designed seven partial ARX constructs spanning the full-length protein (Fig 1A). When these partial recombinant ARX proteins were expressed in HEK293T cells and labelled as above, we determined a positive phosphorylation signal in the N-terminal octapeptide domain region (OP), polyalanine tract 1 & 2 region (PA1&2), polyalanine tract 3 region (PA3) and the homeodomain region (HD) (Fig 1C top panel—arrows). Additional bands on this panel are likely phosphorylated proteins non-specifically captured by the immunoprecipitation process. The presence of each partial protein was confirmed by immunoblotting (IB) using an anti-Myc antibody (Fig 1C, bottom panel). The *ARX* gene encodes a predicted protein of 562 amino acids including 43 serines, 28 threonines and 8 tyrosines (Fig 1D). Given all three residues (serine, threonine, tyrosine) could be potentially modified by phosphorylation, we used anti-phospho antibodies against Myc-ARX overexpressed in HEK293T cells and confirmed that the tyrosine and the threonine residues were not phosphorylated (Fig 1E). However, a positive signal was observed for serine phosphorylation (Fig 1E and S1 Fig). These experiments established that ARX is phosphorylated at multiple serine residues.

### Serine Phosphorylation sites at the N-terminus of ARX

**Fig 2:**
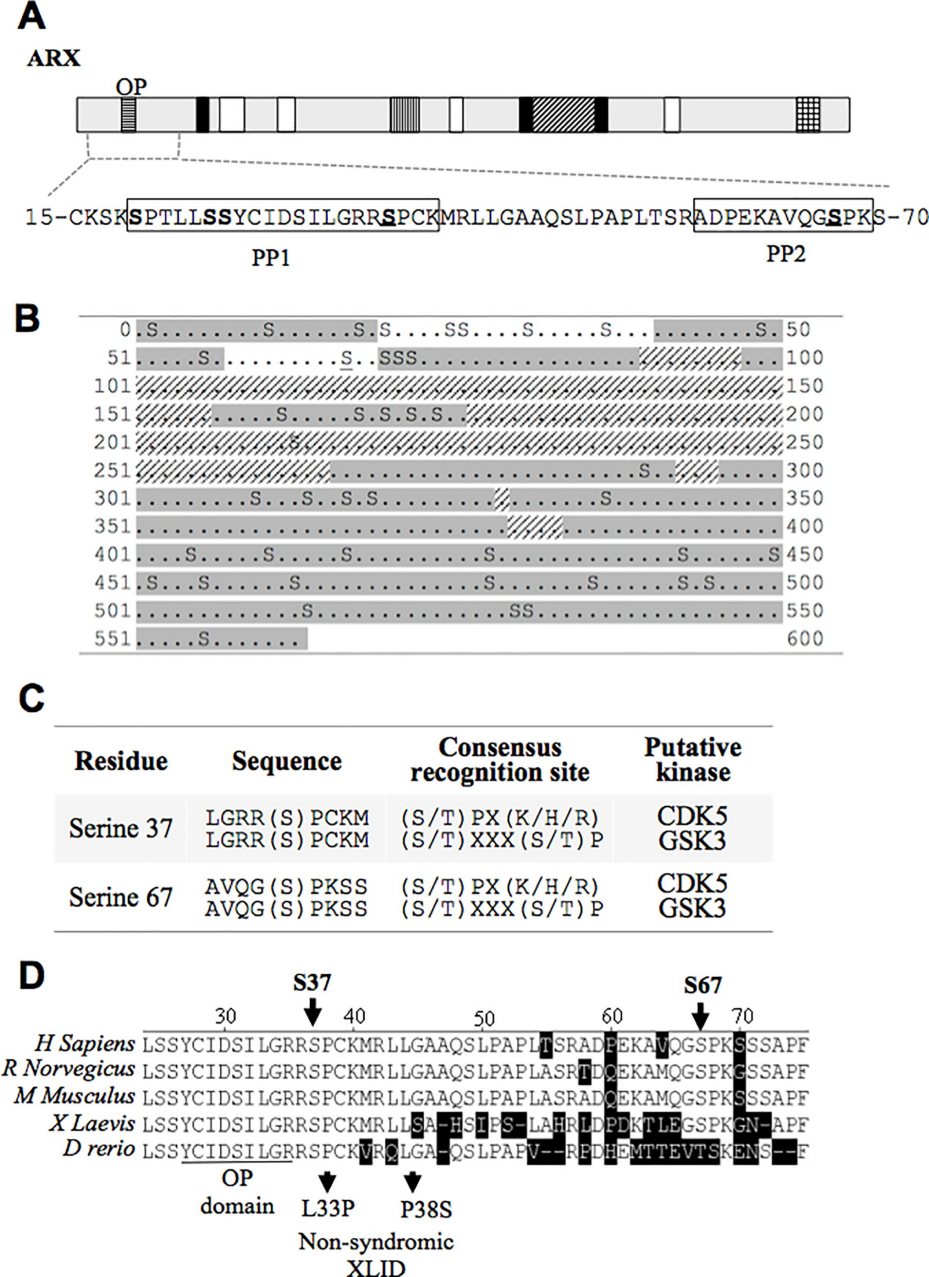
Serine phosphorylation sites in the N-terminus of ARX. (A) The position of phosphopeptides (PP1 & PP2) identified by LC-ESI-IT-MS/MS analysis is indicated by the boxes. The phosphorylated residues S37 and S67 are bold and underlined. (B) Peptide sequence coverage of ARX-WT by MS analysis. Matched peptides in ARX-WT peptide sequence coverage are shown highlight by grey boxes; phosphopeptides and novel phosphorylation site (underlined) detected by MS analysis are indicated in white boxes. Positions boxed in hatched lines were not covered by the MS analysis. (C) NetPhos 3.1 in silico prediction of ARX putative phosphorylation sites. (D) Orthologous protein sequence alignments of ARX protein with non-conserved amino acids shaded in black. Octapeptide domain of ARX is indicated by a dotted lined box. The position of serine phosphorylation sites–S37 and S67 are indicated by black arrows. Below the alignment, naturally occurring ARX mutations causing X-linked ID.

Immunoprecipitated full-length recombinant Myc-tagged ARX protein was isolated using SDS-PAGE and digested with trypsin or chymotrypsin and analysed as a fee for service (Prof Peter Hoffmann, Proteomics Facility, University of Adelaide, Australia) by liquid chromatography-tandem MS (LC-eSI-IT-MS/MS) in a Bruker HCT Ultra 3D-Ion-trap mass spectrometer to identify phosphorylated residues within ARX. Data was analysed with BioTools (Version 3.1, Bruker Daltonics) and reported phosphopeptides were verified by manual inspection of spectra. The fragmented peptides generated by either digestion method were consistent with ARX protein sequence when analysed by MS, indicating the preparations were free of contaminating material. When the Mass Spec analysis from both trypsin and chymotrypsin enzyme digestions were combined, up to 76% of the ARX ORF was successfully mapped (Fig 2B, unmapped areas in grey). Of the 135 residues that were not adequately covered by MS following digestion with either trypsin or chymotrypsin, there was only one serine residue not analysed; serine 213. Therefore phosphorylation at this residue cannot be discounted. Two phosphopeptides (PP1 and PP2) were identified at the N-terminus of ARX (Fig 2A). The phosphopeptide, PP2 spans residues 56-69aa and contains only one modifiable residue, serine 67 (Fig 2A and 2B). This residue is clearly identified as being phosphorylated by the MS peak (S2 Fig). This analysis is in agreement with the HEK293T cell labelling data identifying phosphorylation in the PA1&2 region of ARX (Fig 1C) and a positive signal observed for serine phosphorylation (Fig 1E). The other phosphopeptide, PP1 spans from 20-40aa of the ARX protein and contains 5 potential serine residues that could be modified at residue p.20, p.25, p.26, p.31 and p.37 (Fig 2A and 2B). LC-ESI-IT-MS/MS analysis suggests that serine 20 or 37 are the most likely phosphorylation sites in PP1 (S2 Fig). NetPhos3.1 is a database that predicts putative phosphorylation sites based on consensus kinase recognition sequences [17]. Of the two candidate serine residues in PP1, NetPhos3.1 scores of predicted active kinases for these sites indicated serine 37 was a potential substrate for CDK5 and GSK3. On the other hand, the serine 20 residue prediction scores did not meet the threshold (above 0.5), suggesting that serine 37 is the likely site of phosphorylation in PP1 (Fig 2C and Table 1). To confirm that serine 37 is the site phosphorylated in PP1, alanine substitution of serine 37 was introduced to full-length ARX construct. Full-length Myc-ARX-WT and the ARX-S37A mutant were expressed in HEK293T cells and subjected to two-dimensional gel electrophoresis (2DGE) analysis (S3 Fig). ARX-WT and ARX-S37A mutant proteins displayed multiple protein spots each with a different protein variant pattern when aligned (S3 Fig), indicating substitution of serine 37 with alanine changes the isoelectric properties of the ARX protein. This analysis supports a change in the phosphorylation status and indicates that the serine 37 residue is the site of phosphorylation in PP1. Hence, we identify and confirm that serines at position 37 and 67 of ARX are phosphorylated.

**Table 1:** NetPhos3.1 prediction (scores greater than 0.9) of likely phosphorylation sites in ARX.

aa no#	aa	Predicted Score> 0.9	Predicted Kinase Score > 0.5	Partial Construct	PhosphoAb	MS	*In vivo* labelling
18	S	0.913	CDC2 / 0.51	OP	+	-	+
37	S	0.996	CDK5 / 0.54; GSK3/ 0.51	OP	**+**	**+**	**+**
55	T	0.969		OP	-	-	
67	S	0.995	CDK5 / 0.55; GSK3 / 0.52	OP	**+**	**+**	**+**
170	S	0.901		PA1&2/165-214	+	-	+
174	S	0.998	**PKC** /**0.79;** RSK / 0.6	PA1&2/165-214	+	-	+
223	T	0.941		PA3	-	-	
290	S	0.996	CKII / 0.60; CKI / 0.53	PA3	+	-	+
317	S	0.912	CKII / 0.69; CDC2 / 0.53	HD	+	-	+
319	S	0.987	CKI / 0.52; CKII / 0.51	HD	+	-	+
411	S	0.941	p38MAPK/0.58;GSK3/ 0.51	HD	+	-	+
530	S	0.996		OAR	+	-	-
531	S	0.981		OAR	+	-	-
557	T	0.987		OAR	-	-	

### Alternative strategies to identify predicted sites of phosphorylation in the ARX protein

**Fig 3:**
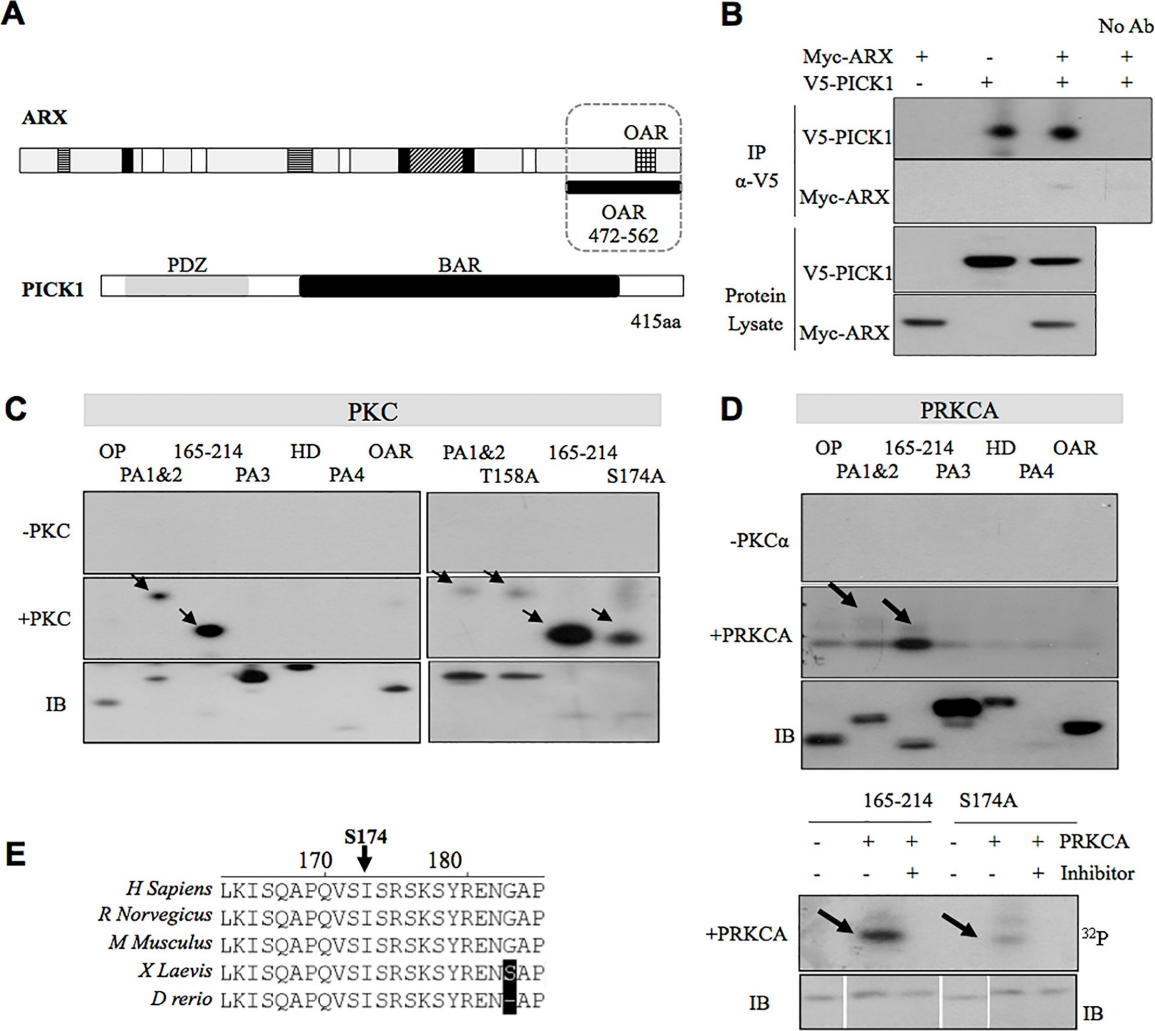
ARX is phosphorylated by PRKCA through the interaction with PICK1 in mammalian cells. (A) The C-terminal partial construct of ARX (OAR containing region in dotted box) identified PICK1 as an interacting protein by Y2H assay and this interaction was confirmed by Co-IP in (B). HEK 293T cells transfected with *Myc-ARX* and *V5-PICK1* constructs were lysed and proteins immunoprecipitated (IP) with rabbit anti-V5 antibody. Protein samples were separated by SDS-PAGE and analysed for the presence of Co-IP Myc-ARX by immunoblotting with mouse anti-Myc horseradish-peroxidase (HRP) conjugated antibody (bottom lane of top panel). V5-tagged PICK1 in the immunoprecipitate is shown in top panel. Myc-tagged ARX and V5-tagged PICK1 proteins are shown in the protein lysate in the bottom panel. Cells transfected with *Myc-ARX* alone or *V5-PICK1* alone included as negative controls. A representative blot is shown from two independents experiments. Partial ARX constructs as described for Fig 1B used in an *in vitro* kinase assay with the addition of (C) PKC pool (top panel) (D) or PRKCA (left panel). (E) Orthologous protein sequence alignments of ARX protein with non-conserved amino acids shaded in black. The position of serine phosphorylation site S174 is indicated by a black arrow. Separate blots are boxed with a black outline. Separate blots are boxed with a black outline. Spliced lanes on the same blot are indicated by white spaces.

Our *in vivo* labelling data (Fig 1) indicates that there are likely sites of phosphorylation that were not detected by mass spectrometry, even when employing an additional strategy of phosphopeptide enrichment (using a magnetic bead-based immobilised metal affinity chromatography capture kit). Taking an unbiased approach we analysed the ARX protein sequence using *in silico* prediction software, NetPhos3.1. The protein sequence in FASTA format was used as input for NetPhos3.1 software. ARX has 11 serine, and 3 threonine sites predicted to be phosphorylated with a score greater than 0.9, which suggests very likely phosphorylation sites (Table 1). The anti-phosphoantibody immunoblot data in Fig 1E indicates that tyrosine and threonine residues of recombinant full-length ARX are not phosphorylated in HEK293T cells (Table 1- dark grey). Rows highlighted in light grey indicate no phosphorylation signal by *in vivo* labelling assay of partial recombinant proteins. Combining the *in silico* analysis and the *in vivo* labelling data and the phosphoantibody data we refined the predicted phosphorylation sites to a possible 9 serine residues across the ARX protein (Table 1). Two of these sites were those confirmed by MS analysis; residues S37 and S67 (Table 1-highlighted in green). Both Serine 37 and 67 are predicted substrates of CDK5 and GSK3 (Fig 2C and Table 1). Hence, there were 7 remaining serines with a positive signal in the *in vivo* labelling assay but were not detected as phosphorylated by MS analysis when overexpressed in Hek293T cells. Using our *in silico* approach, possible active kinases for each of these sites were identified using a prediction score above 0.5 (the prediction score is a value in the range [0.0–1.0]; with a score above 0.5 indicating a positive prediction). The kinase listed for Serine 174 was PKC (Table 1- highlighted in yellow). Interestingly, a yeast-2 hybrid screen of a human fetal brain cDNA library using the human ARX *Aristaless* domain (472-562aa) as a bait identified protein interacting with C kinase 1 (PICK1) (GeneBank: NM_012407) (Fig 3A) as a novel protein partner. PICK1 contains a PDZ domain (Fig 3A) that is known to interact with PKC, in particular, the PRKCA isoform, at its C-terminal PDZ-binding domain. Hence, the analysis of predicted sites and kinases indicates that S174 of ARX may be subject to phosphorylation by PRKCA, facilitated by ARX binding to PICK1. The remaining 6 serines may represent additional sites of phosphorylation in ARX but were not pursued in the current investigation.

### ARX is phosphorylated at S174A by PRKCA

PRKCA is known to interact with PICK1 at its C-terminal PDZ-binding domain (Fig 3A). This interaction regulates specific subcellular localisation of PRKCA to phosphorylate its target protein [16]. The interaction of ARX and PICK1 led us to predict that ARX might be phosphorylated at S174 by PRKCA and that this modification, potentially regulates ARX activity. To examine whether PRKCA directly phosphorylates ARX, we first performed *in vitro* kinase assays using a pool of purified protein kinase C (PKC) enzymes. Overexpressed full-length Myc-ARX immunoprecipitated from HEK293T cells or ARX produced using a cell-free *in vitro* transcription-translation system (cell-free Xpress-tagged full-length ARX) were used as substrates. The enzymes used in this kinase assay were a pool of PKC isozymes isolated from rat brain, consisting mainly of classical PKC isozymes (PRKCA, PRKCB and PRKCG). Using this approach, the cell-based Myc-ARX was phosphorylated upon addition of the PKC enzyme pool (S4 Fig; top panel). The inclusion of a PKC inhibitor led to the attenuation of the phosphorylation signal, confirming the phosphorylation of ARX by PKC. A known PKC substrate, neurogranin (NGRN) [18] served as a positive control in the assay (S4 Fig). Interestingly, when we used the Xpress-tagged ARX substrate in this assay (S4 Fig), background bands of the IP antibody and sepharose complex were observed but no specific band correlating to ARX protein was detected. Hence, cell-free ARX was not phosphorylated by PKC (S4 Fig). We cannot rule out that cell-free ARX may not have been correctly folded or modified to obtain the necessary tertiary structure required for PKC modification by this assay. Despite this, our data suggest that a factor(s) present in mammalian cells that co-immunoprecipitated with ARX may be important for phosphorylation of ARX by PKC. One such factor may be the novel protein partner PICK1.

Co-immunoprecipitation was used to confirm the interaction of ARX and PICK1. Myc-tagged ARX and V5-tagged PICK1 were co-expressed in HEK293T cells. When V5-tagged PICK1 was immunoprecipitated (IP) from total cell lysates using a monoclonal anti-V5-HRP antibody, Myc-tagged ARX (~62 kDa) was detected by monoclonal anti-Myc antibody in this precipitate (Fig 3B). Protein lysates from cells transfected with Myc-ARX alone and V5-PICK1 alone were included as controls. Specific IP of overexpressed V5-tagged proteins was confirmed by the negligible protein detected in the no anitbody lane, demonstrating very low levels of nonspecific binding of polyalanine rich ARX to protein A sepharose beads [19]. Taken together, our data show that ARX interacts with PICK1 in mammalian cells.

To confirm that S174 is the specific residue phosphorylated by PKC we used the Myc-tagged partial proteins spanning ARX (Fig 1A) and demonstrate that PKC phosphorylates the PA1&2 and 165–214 regions (Fig 3C, indicated by arrows in top panel). The presence of each partial protein was confirmed by immunoblotting with an anti-Myc antibody. The levels of detectable protein for 165–214 were very low. Despite this, its phosphorylation signal by the radioactive assay was evident (Fig 3C, lane 3 middle panel). Although serine 174 is present in both PA1&2 and 165–214 partial proteins, it is the very last reside in the PA1&2 (61-174aa) and as such lacks the necessary surrounding residues to form the PKC consensus recognition sequence, making phosphorylation at this site in the PA1&2 partial protein unlikely. As expected, no phosphorylation signal was observed in the absence of PKC enzyme (Fig 3C). Although modification at threonine residues was unlikely given the data in Fig 1E, we chose to interrogate the phosphorylation by PKC at two sites predicted by *in silico* analysis, with alanine substitution in place of threonine at position 158 in PA1&2 and serine at position 174 in the 165–214 partial proteins. In the absence of PKC enzyme, no phosphorylation was detected in any sample (Fig 3C). In the presence of PKC enzyme, the phosphorylation signal of phospho-null mutant p.S174A was markedly reduced compared to the corresponding ARX-WT partial protein (165–214) (Fig 3C, right top panel). This reduction in phosphorylation signal was in the context of similar levels of protein in both the ARX-WT and p.S174A samples (Fig 3C, right bottom panel lane 3 and 4). This result confirms that serine at position 174 is modified by PKC. However, no difference in the phosphorylation signal was observed when the PA1&2 protein with p.T158A mutation was assayed compared to the wild-type equivalent (Fig 3C, right middle panel lane 1 and 2), showing that this threonine is not PKC modified. This outcome agrees with our previous results demonstrating that threonine residues in ARX are unlikely to be phosphorylated (Fig 1E).

PICK1 is a protein that plays a key role in PKC phosphorylation and links PRKCA to its targets [16]. Given that we have identified PICK1 as a novel protein partner of ARX, we wanted to investigate if the classical PRKCA was responsible for this phosphorylation of ARX, particularly at serine 174. We began by investigating if PRKCA interacts with ARX, using Co-IP. Myc-ARX and HA-PRKCA were co-transfected into HEK293T cells. When the HA-PRKCA was immunoprecipitated using anti-HA antibody we detected Myc-ARX co-immunoprecipitating when immunoblotted with anti-Myc antibody (S5 Fig). Next, we substituted PRKCA as the enzyme in place of the commercially available PKC enzyme pool. A clear signal is again noted in the 165–214 partial protein (Fig 3D, middle panel). Similar to the previous assay, the phosphorylation signal of mutant p.S174A is attenuated compared to the corresponding wildtype 165–214 partial protein (Fig 3D, right panel). The partial ARX proteins were confirmed by immunoblot using anti-Myc antibody. Our data demonstrates that serine 174 is the site modified by PRKCA in partial protein 165–214. Serine S174 is conserved across multiple ARX orthologs as shown in (Fig 3F).

### The function of ARX as a transcriptional regulator requires phosphorylation

To determine the impact of serine phosphorylation on the function of the ARX protein, phospho-null substitutions were generated in which the serine residues identifed to be phosphorylated were replaced with alanines. Using a dual-luciferase reporter assay described in our previous work [20, 21] we measured the impact of these mutations on the interaction with an orthologous Arx-binding site in the enhancer region of *Lmo1* [22] inserted upstream of an SV40 promoter driving luciferase expression. Using this assay, we demonstrate that phosphorylation of serine residues identified in this study does not affect ARX transcriptional activity in HEK293T cells (S6 Fig). This reflects that phosphorylation at these residues does not negatively impact the binding of the ARX homeodomain to the identified transcription factor binding motif in this *in vitro* setting [22]. However, ARX is known to be able to act as both an activator and repressor of gene expression. Hence, we chose to determine the functional consequences of phosphorylation of the ARX protein using an unbiased approach of transcriptome-wide analysis. Two phospho-null mutants were included in the analysis, ARX-S174A and a double mutant ARX-S37AS67A, compared to untransfected cells and ARX-WT controls. We chose to use alpha TC cells, a mouse pancreas-derived cell line in which Arx is vital for the establishment and maintenance of glucagon-producing alpha cells. Although the endogenous levels of *Arx* expression within the pancreas are tightly regulated, and lower than expression within selected brain tissue, we reasoned that these cells would contain the necessary cellular machinery required for *Arx* transcriptional activity. Transcriptomes of cells transfected with ARX-WT and phospho-null mutants were compared to transcriptomes of the untransfected cells to determine specific gene expression changes triggered by phosphorylated/phospho-null ARX. In all experiments, we achieved similar transfection efficiencies as determined by immunofluorescence. Individual genes were considered to be differentially expressed with a significance threshold of log2FC<±1, P>0.05 applied to the analysis. Validation of RNASeq analysis showed a correlation of deregulation detected by qRT-PCR (S7 Fig). To investigate genes deregulated in alpha TC cells either expressing ARX WT, or ARX phosphorylation mutants we compared functional enrichment analysis of gene ontology (GO) terms for biological processes (S1 Table), cellular component (S2 Table) and molecular function (S3 Table). Genes with a log2 fold change with +/->1 cutoff value were used as the input into EnrichR (23). This analysis highlights some of the pathways that may be deregulated in response to the ablation of different phosphorylation sites, however, due to the modest list of deregulated genes used as the input for analysis we saw no significant enrichment of terms.

**Fig 4:**
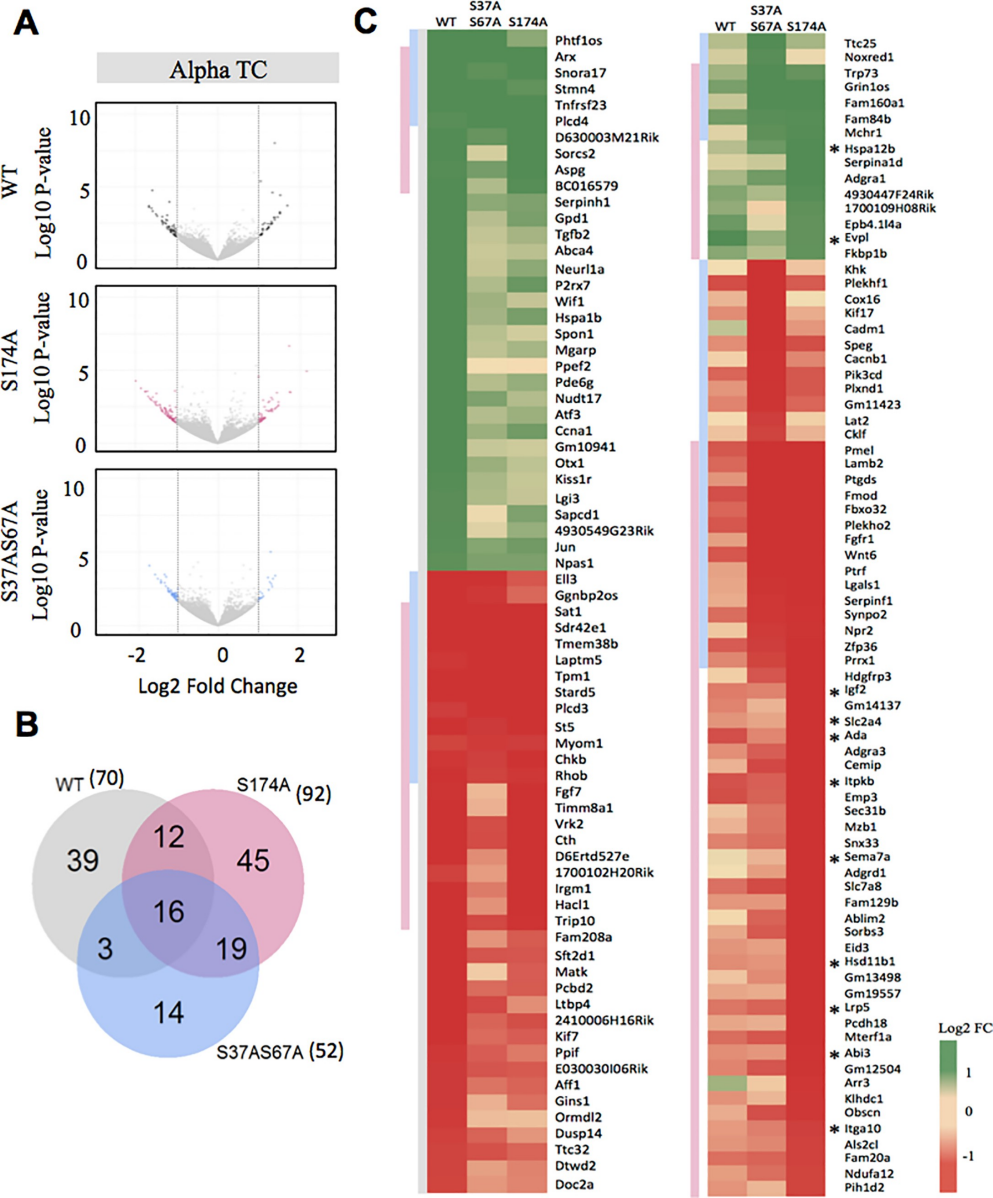
Transcriptional changes due to the loss of phosphorylation sites in ARX. (A) RNASeq analysis looking at the transcriptional changes by ARX-WT and two phospho-null constructs, ARX-S174A and ARX-S37AS67A overexpressed in alpha TC cells. The x-axis represents log2 expression fold-change in UT vs. WT/S174A/S37AS67A and the y-axis represents P-value (-log10). Up-regulated genes (P-value < 0.05 & log2-FC > 1.0) and down-regulated genes (P-value < 0.05 & log2-FC < -1) are shown in colour. (B) The numbers and overlap of the total number of significantly deregulated genes in RNASeq analysis using alpha TC cells by ARX-WT (grey), ARX-S174A (pink) and ARX-S37AS67A (blue). (C) Heatmap of the significantly deregulated genes (rows) by ARX-WT, ARX-S174A and ARX-S37AS67A (columns). The left panel lists the genes significantly deregulated by ARX-WT and the corresponding transcriptional changes for the same genes by ARX-S174A and ARX-S37AS67A. The right panel highlights the additional genes deregulated by either phospho-null mutant ARX-S17A or ARX-S37AS67A or both indicated by the pink or blue bars on the left on the heatmap. Genes with a PAX4 motif within the promoter region are indicated by a star.

Overexpression of ARX–WT resulted in 70 genes significantly deregulated compared to the untransfected alpha TC cells (Fig 4A) (Data has been submitted to Gene Expression Omnibus under the GEO accession number: GSE105113). Of the 70 genes, we saw 53% of these were downregulated when compared to untransfected alpha TC cells. When ablation of phosphorylation sites p.S174A or p.S37A67A in ARX were tested, there were 92 and 52 genes significantly impacted, respectively (Fig 4B). Of the genes impacted by the phospho-null mutants, 75% were downregulated for both ARX-S37AS67A and ARX-S174A. Next, we compared the 70 genes that were changed in ARX-WT (black) alpha TC cells to the genes deregulated in the phospho-null mutants (p.S174A, pink and p.S37AS67A, blue). This analysis shows that the ablation of phosphorylation at these sites lead to a partial loss of function, with 73% (51/70) and 60% (42/70) of genes no longer being significantly deregulated in ARX-S37AS67A and ARX-S174A, respectively (Fig 4C, left panel). Loss of phosphorylation at the sites tested resulted in significantly altered expression of an additional 45 genes (82% with reduced expression) for ARX-S174A and 14 genes for ARX-S37AS67A and another 19 genes for both mutations that were not impacted by ARX-WT overexpression (Fig 4C, right panel). Of the 45 genes significantly altered by the phospho-null ARX-S174A mutant, using Enrichr, a enrichment analysis tool [23], we saw a significant enrichment of genes (Adjusted p-value = 0.02948) with a PAX4 transcription factor DNA binding motif within the promoter region (indicated by a star in Fig 4C, right panel) [23].

### The function of ARX as a transcriptional regulator requires the C-terminal region that binds and co-localises PICK1

**Fig 5:**
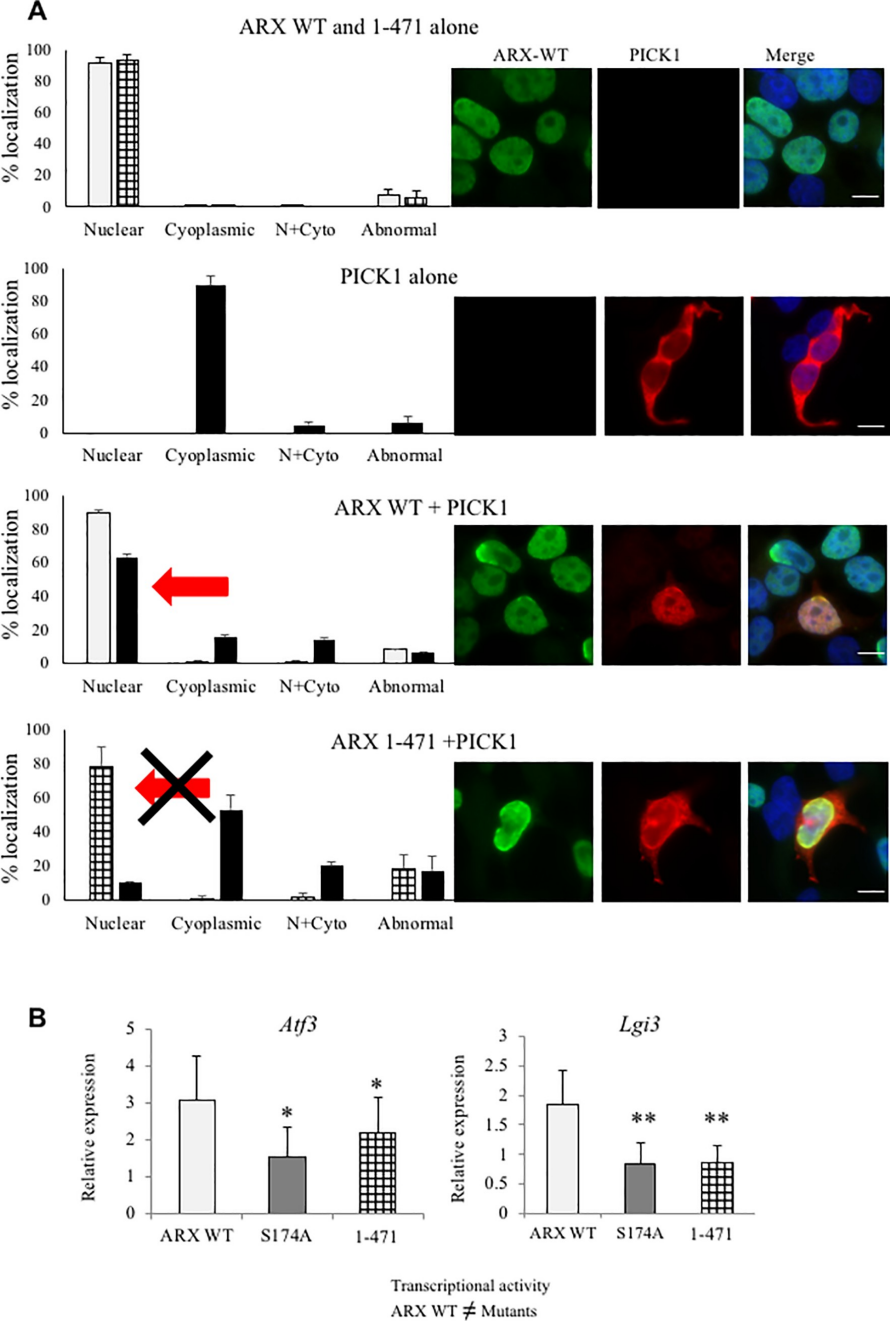
C-terminal region of ARX is required to bind and co-localise PICK1 and aspects of transcriptional activity. **A)** Left hand panel shows graphs depicting the percentage of transfected cells with nuclear, cytoplasmic, both nuclear and cytoplasmic or abnormal subcellular localisation for ARX-WT (grey), ARX 1–471 (cross hatched), PICK1 (black) and when co-transfected. The percentage of cells was determined from ARX-WT alone (n = 850); PICK1 alone (n = 252), ARX 1–471 alone (n = 514), and co-transfected with ARX-WT and PICK1 (n = 260) and ARX 1–471 and PICK1 (n = 228) from at least two separate transfection reactions, 24 hours post transfection. The right-hand panel shows pictomicrographs of the predominant subcellular localisation for each construct, with ARX proteins detected in green (Alexa-488) and PICK1 detected in red (Alexa-555), with the merged image on far right. Scale bar = 10μm. **B)** qRT-PCR analysis of changes to deregulated gene expression with ARX WT, S174A phospho-null mutant and the C-terminal truncated ARX 1–471 overexpressed in alpha TC. cDNA was prepared from total RNA extracted from transfected alpha-TC. Expression values measured by qRT-PCR were normalised to reference gene *Beta-Actin*. Individual graphs for each gene are shown relative to expression in mock transfected alpha-TC cells set at 1.0, with ARX WT (pale grey), S174A phospho-null mutant (dark grey) and C-terminal truncated 1–471 (cross hatched). Error bars indicate standard error of the mean from replicate cDNA samples prepared from repeat transfections. *P<0.05, **P<0.01 (one-tailed t-test of S174A or 1–471 mutant compared to ARX WT).

To examine the role of PICK1 binding impacting the transcriptional activity of ARX, we removed the c-terminal region of ARX that was initially used to establish PICK1 as a binding partner. Hence, we introduced a stop codon at p.R472X lead to truncation of the protein at p.471. This c-terminal truncated protein displayed the same subcellular nuclear localisation pattern as the full length ARX WT protein (Fig 5A). This was not unexpected, with the homeodomain and flanking nuclear localisation sequences of ARX known to be necessary and sufficient to localise the protein to the nucleus [20]. When PICK1 was co-transfected with ARX-WT there was no change to the localisation pattern of the ARX protein. In contrast, PICK1 protein shifts from a predominate cytoplasmic localisation (89.5%) when overexpressed alone, to being co-located with ARX in the nucleus in co-transfected cells (15% cytoplasmic vs 63% nuclear) (Fig 5A). However, this shift of PICK1 protein to the nucleus was not observed when co-transfected with the C-terminal truncated ARX 1–471 protein (Fig 5A). Instead, the majority of co-transfected cells display PICK1 still in the cytoplasm (53%) with increased numbers of cells with either the PICK1 protein localised more diffusely in both the nucleus and cytoplasm (20%) or in cells with abnormal protein localisation (17%) of both PICK1 and ARX 1–471 protein. Next, we compared the impact of the c-terminal region of ARX binding to PICK1 on the transcriptional activity of the ARX protein. ARX WT overexpression in alpha TC cells results in transcriptional activation of *Aft3* and *Lgi3* genes by RNASeq analysis. This activation is confirmed by qRT-PCR from multiple transfections. Although the phospho-null S174A mutant does not abolish the overall transcriptional activity of ARX protein, the post-translation modification at S174 is required for adequate transcriptional activation of these specific gene targets by qRT-PCR (Fig 5B). The outcome agrees with the RNASeq analysis for S174A (Fig 4C). Interestingly, we demonstrate that the truncation of the c-terminal region of ARX that is required to bind and co-localise PICK1 displays a loss of transcriptional activation comparable to the S174A mutant in relation to the ARX WT activity for *Aft3* and *Lgi3* genes tested (Fig 5B).

## Discussion

In this study, we have identified ARX as a multisite phosphoprotein. By LC-eSI-IT mass spectrometry, we were able to identify serine 37 and serine 67, both in the N-terminal region of the protein, as phosphorylation sites. Additional phosphorylation sites were indicated experimentally, but could not be resolved at the present time. We identified a novel ARX protein partner, PICK1, by yeast 2-hybrid and co-immunoprecipitation, which implicated PRKCA as the ARX phospho-modifying enzyme. We subsequently demonstrated that PRKCA phosphorylates ARX at multiple sites, including serine 174 as the major PRKCA phosphorylation target. We demonstrate that phospho-null mutants do not affect ARX transcriptional activity in HEK293T cells using an *in vitro* luciferase reporter assay. However, our study shows that phosphorylation at these three sites is very likely required for correct transcriptional activity of the ARX protein in a correct cellular context. Using a transcriptome-wide approach to analyse gene expression in pancreatic alpha TC cells we show that compared to untransfected cells, the significantly regulated gene expression with overexpression ARX-WT was markedly reduced with the double phospho-null mutant Ser37Ala+Ser67Ala and Ser174Ala. Generating a c-terminally truncated mutant of ARX, we confirm that the c-terminal region of ARX is required to bind PICK1 and show this binding causes a shift in PICK1 subcellular localisation to the nucleus to co-locate with the ARX protein. Moreover, we demonstrate that the c-terminal truncated mutant of ARX gives rise to the same loss of transcriptional activation as S174A mutant using qRT-PCR analysis of targets identified in RNA-Seq analysis. Our study demonstrates for the first time that ARX is phosphorylated at several sites and that phosphorylation is important for aspects of transcriptional activity.

Our data establishes that there are at least three novel phosphorylation sites across ARX, serines 37, 67 and 174. The two serine residues at the N-terminal region of the protein were identified using a LC-eSI-IT mass spectrometer. Detection by this method implies that a reasonable proportion of the overexpressed ARX-WT protein was post-translationally modified at these sites. Both are predicted to be substrates of CDK5 or GSK3. Cyclin-dependent kinase 5 (CDK5) ubiquitously expressed in mammalian tissues [24]. Within the nervous system, CDK5 is involved in a range of functions including neuronal migration. Glycogen Synthase Kinase-3 (GSK3) is a promiscuous protein kinase known to phosphorylate a diverse range of substrates. Almost all GSK3 substrates require a priming phosphorylated residue to be 4 amino acids C-terminal to the Serine/Threonine phosphorylated by GSK3, although unprimed GSK3 mediated phosphorylation has also been reported [25, 26]. There are no Serine or Threonine sites 4 amino acids C-terminal of serine 37 in ARX. We cannot rule out that a priming event may occur 4 amino acids C-terminally of serine 67 at serine 71 in ARX required for GSK3, however, we were not able to detect any priming event at this site by the current analysis.

The third phosphorylation site, serine 174 was identified and demonstrated to be a novel site phosphorylation by PRKCA. PKC translocates to different subcellular sites, generally from cytosolic to membrane, and phosphorylates and regulates its target proteins as a consequence of this translocation. Identifying this kinase modifying ARX at serine 174 was achieved through our identification of PICK1 as a novel interacting partner. As PICK1 is known to have a key role in PRKCA phosphorylation by linking PRKCA to its target proteins, we predict that the interaction of PRKCA with ARX to modify serine 174 is enhanced by the interaction with PICK1. Interestingly, PICK1 is expressed in many tissues with the highest expression found in the brain, followed by the testis (15). These are both sites in which ARX is also expressed [27, 28]. PICK1 is known to be localised to the cytoplasmic region of cells, in particular, the perinuclear region and associates with the mitochondria. PICK1 contains both a PDZ domain and a BAR domain, enabling multifunctional capacity to bind to a large selection of proteins, including more than one at a time. The PICK1-PRKCA interaction is formed between the PDZ domain of PICK1 and PDZ ligand of PRKCA [16]. The bait construct used to identify PICK1 as a binding partner of ARX consisted of the C-terminal portion of the ARX protein including the aristaless (OAR) domain. ARX belongs to a subset of paired class homeoproteins which are characterised by the presence of this conserved domain located in the carboxyl-terminal region known as the OAR domain. The presence of the aristaless domain in a subset of Paired-like homeodomain proteins and the invariable C-terminal location suggest that it has a molecular function that is inherently and directly related to the way these homeoproteins exert their function. A function as an activation domain in the Otp protein was proposed originally [29], but more recent studies including both *in vitro* and *in vivo* data suggests that the aristaless domain functions to attenuate transcriptional activation [10, 30, 31]. It has been proposed that intramolecular interactions between the aristaless domain and the N-terminus of these proteins lead to a protein conformation associated with a relatively inactive state of these transcription factors. Conceivably, intermolecular interactions *in vivo* would unfold the protein leading to its activation. Our finding of ARX-PICK1 binding through the C-terminal region of the ARX protein, including the OAR domain and PRKCA phosphorylation of ARX through PICK1, a scaffold protein, allowing PRKCA to access the phosphosite of ARX we predict drives this conformation change. Interestingly, a number of missense mutations across this C-terminal portion of the *ARX* gene including the *Aristaless* domain lead to Early Infantile Epileptic Encephalopathy (MIM#308350) or in one case the severe brain malformation phenotype of lissencephaly, X-linked 2 (MIM#300215). Further studies are required to assess if these missense mutations negatively impact on the capacity of the ARX protein to be phosphorylated and alter transcriptional capacity contributing to disease.

Post-translational modification of transcription factors can alter the dynamics by which they function [32]. Phosphorylation in particular, can have multiple consequences, including interference of the phospho-group with DNA binding leading to inactivation of the transcription factor, increased protein stability and interaction with putative partners, and consequently DNA binding and target gene activation [33–35]. In HEK293T cells we showed ablation of each serine site identified as modified by phosphorylation did not result in marked changes to the normal subcellular localisation of the protein (overexpression studies). Nor was there a loss of transcriptional capacity of our phospho-null constructs compared to ARX-WT using a luciferase reporter assay consisting of three copies of the orthologous region identified as an ARX-binding site in the enhancer region of Lmo1 upstream of SV40-luciferase. This outcome indicates that phosphorylation of the ARX protein at these sites is not required for the homeodomain of ARX to bind to DNA motif containing ‘TAATTA’, at least in this *in vitro* settting. As these phosphorylation sites fall outside of the homeodomain it is likely that each site may influence the affinity and selectivity of protein-DNA and/or protein-protein interactions. Hence, each site modified may contribute to altering transcriptional activity differently through promoting association with distinct molecular partner/promoter regions.

RNASeq was used to determine the transcriptional consequences of two phospho-null constructs, p.S37AS67A and p.S174A. When phosphorylation at these sites was blocked, the transcriptional capacity of ARX in the alpha TC pancreatic cell line in which Arx is known to have a role [7, 36], there was a considerable number of deregulated genes in mutant vs ARX-WT analysis. When the c-terminal region of ARX was truncated, the capacity to bind and co-localise PICK1 into the nucleus of cells with ARX protein was lost, and a similar loss of transcriptional activity as the S174A mutation was demonstrated by qRT-PCR. This strongly supports the role of PICK1 binding to the C-terminal region of ARX in subsequent phosphorylation at S174 by PRKCA required for transcriptional activity of the ARX protein on selected gene targets.

The phosphorylation state and the functional relevance of this modification in other cell types in which ARX plays a biological role is required, particularly in the brain. Hampering these efforts are the difficulties in isolation of adequate amounts of endogenous ARX required for analysis. This is due in part to the limited expression pattern of this transcription factor in the brain during early embryonic development coupled to the lack of a high specificity antibody against endogenous ARX/Arx, despite many antibodies generated to date. Ideally, generation (by CRISPR/Cas9 technology) of a mouse line in which the Arx locus is manipulated to incorporate a HA or Flag tag is required as a viable strategy to target the tagged-endogenous Arx protein by validated antibodies against the tag. This approach would provide an opportunity to investigate the role of Arx as a critical transcription factor in developmental processes, particularly in the brain, as well as the pancreas and reproductive organs.

ARX is known to be able to act as both an activator and repressor of gene expression. ARX-WT activity in alpha TC cells resulted in an even distribution of genes with increased (activated) and decreased (repressed) levels of expression. We saw an increase in the proportion of genes remaining repressed when alpha TC cells were transfected with the phospho-null mutants. We also saw additional targets repressed in the ARX-S174A mutant further supporting ARX-PICK1-PRKCA relationship for the activation of transcriptional activity. Pathway analysis of the genes specifically deregulated by ARX-S174A highlighted an enrichment of genes with a PAX4 motif upstream. ARX and PAX4 are drivers of different endocrine precursor cell fates. Indeed, ARX is involved in specifying the α-cell fate whereas Pax4 promotes the β and δ cell lineages [7, 11, 37]. These two factors were found to mutually inhibit each other’s transcription and to display antagonistic activities for proper endocrine cell lineage allocation [11]. Lineage-tracing studies have shown that α-cells can serve as progenitors of β-cells and upon Arx inactivation, α-cells can be continuously regenerated and converted into β-like cells. The additional loss of Pax4 does not impact these processes, suggesting that Arx is the main trigger of α-cell-mediated β-like cell neogenesis [38]. Therefore posttranslational modification of ARX may be an important switch driving between α and β cell fates. The present study establishes ARX as a multisite phosphoprotein. Our study suggests that ARX phosphorylation may have biological and in extrapolation, neurodevelopmental relevance. Further investigations to establish the *in vivo* modification of the ARX protein across relevant tissues and developmental time points are warranted.

## Materials and methods

### Cloning of ARX expression constructs

*ARX* constructs were cloned into Gateway-compatible pCMV-myc vector (BD-Biosciences) with an N-terminal Myc-tag or Gateway-compatible pEXP1-DEST vector with an N-terminal Xpress-tag (Invitrogen). All clones were sequenced to exclude introduced errors and the concentration determined spectrophotometrically. The *ARX* constructs include full-length *ARX* (ARX) with a 1686 bp human *ARX* cDNA (GenBank: NM_139058.2) and seven partial *ARX* constructs spanning the complete *ARX* open reading frame. Each partial construct was named after the functional domain or amino acids (aa) expressed: OP (1–69 aa with the octapeptide domain), PA1&2 (61–174 aa with polyalanine tract 1 and 2), 165–214 (165–214 aa with no functional domain), PA3 (215–302 aa with polyalanine tract 3), HD (303–431 aa with the homeodomain), PA4 (424–474 aa with polyalanine tract 4) and OAR (472–562 aa with the *Aristaless* domain).

Serine residues identified by mass spec (S37 and S67) and *in silico* predicted ARX phosphorylation residues, threonine 158 and serine 174 were mutated in partial and full-length *ARX* constructs by site-directed mutagenesis (Stratagene) following manufacturer’s instructions. To improve the outcome of the mutagenesis reaction in residues located adjacent to GC-rich regions of *ARX*, additional primers (an *ARX* primer and a vector primer) with the same orientation as the mutagenesis primer were added. Alanine (A) substitutions were introduced to abolish the most likely phosphorylation sites (phospho-null mutants) including; two changes c.109A>G and c.110G>C to achieve p.S37A (ARX-S37A), c.199T>G leading to p.S67A (ARX-S67A), and all three combined to give an ARX-S37AS67A double mutant; c.472A>G leading to p.T158A (ARX-T158A) and c.520T>G leading to p.S174A (ARX-S174A). To test the role of the C-terminal binding of PICK1 on ARX transcriptional activity, a c.1414C>T change was generated to lead to a stop codon at p.R472X. All primers used for mutagenesis were HPLC purified.

### Cell culture and transient transfection

Human embryonic kidney 293T (HEK293T) cells were maintained in Dulbecco’s modified Eagle’s medium (DMEM) supplemented with 10% (v/v) fetal calf serum and 100 U/ml sodium penicillin and 100 μg/ml of streptomycin sulphate in 5% CO_2_ at 37°C. Mouse pancreatic alpha (αTC) cells were maintained in DMEM and Nutrient Mixture F-12 (ThermoFisher) supplemented with 10% (v/v) fetal calf serum and 20 U/ml sodium penicillin and 20 μg/ml of streptomycin sulphate in 5% CO_2_ at 37°C. Prior to transient transfection, HEK293T and αTC cells were plated at 8x10^5^ cells per well in a 6-well plate or 4x10^5^ cells per well in a 12-well plate and grown overnight. Next day, 0.5 or 1.0 μg of plasmid DNA per construct was transiently transfected into HEK293T cells using Lipofectamine 2000 (Invitrogen) and αTC cells using Lipofectamine LTX (Invitrogen) following manufacturer’s instructions.

### In cell labelling assay

24 hours post-transfection, HEK293T cells were cultured with ^32^P orthophosphoric acid at 0.5 mCi/ml (as the only source of phosphate) for four hours in 5% CO_2_ at 37°C. Cells were lysed and immunoprecipitated with anti-Myc antibody (Santa Cruz) and cell lysates were subjected to 12% SDS-PAGE gels and the separated proteins were transferred from the gel to a nitrocellulose membrane. Phosphorylated ARX proteins were detected by film autoradiography.

### Mass spectrometry

Protein band corresponding to ~62kDa were excised from the gel, destained and subjected to trypsin or chymotrypsin digestion and resulting peptide extracts were reduced by vacuum centrifugation. LC-eSI-IT MS/MS was performed using an online 1100 series HPLC system (Agilent Technologies) and HCT Ultra 3D-Ion-Trap mass spectrometer (Bruker Daltonics). The LC system was interfaced to the MS using an Agilent Technologies Chip Cube operating with a ProtID-Chip-150 (II), which integrates the enrichment column (Zorbax 300SB-C18, 4 mm, 40 nL), analytical column (Zorbax 300 SB-C18, 150 mm x 75 μm), and nanospray emitter. Fragmented ARX peptides were separated by reverse phase HPLC using C-18 column and 0–30% acetonitrile gradient. MS and MS/MS spectra were subjected to peak detection and de-convolution using DataAnalysis (Version 3.4, Bruker Daltronics). Compound lists were exported into BioTools (Version 3.1, Bruker Daltonics) and submitted to Mascot (Version 2.2) LC-ESI-IT-MS/MS analysis was performed as a fee for service by Adelaide Proteomics Centre, University of Adelaide, Australia.

### Cell-based protein expression and purification

Transfected cells were harvested at 24 hours post-transfection and cell lysates were prepared using extraction buffer 1 for *Myc-ARX* transfected cells (120 mM NaCl, 50 mM Tris-HCl (pH 8.0), 0.5% NP-40 (v/v), 1X protease inhibitor cocktail (Sigma), 1 mM Na_3_VO_4_, 1mM NaF, 1mM PMSF) or extraction buffer 2 for *HA-PKC* transfected cells (50 mM HEPES (pH7.5), 150 mM NaCl, 1 mM EDTA, 2.5 mM EGTA, 10% glycerol, 0.1% Tween-20, 1X protease inhibitor cocktail (Sigma), 1 mM PMSF, 1 mM NaF, 1 mM Na_3_VO_4_, 50 mM β-glycerolphosphate). Myc-ARX or HA-PKC proteins were immunoprecipitated overnight at 4°C from total cell lysates using an anti-Myc antibody (Santa Cruz, 9E10) or an anti-HA antibody (Sigma, H9658) on protein A sepharose (GE Healthcare). Next day, sepharose was washed three times with wash buffer (120 mM NaCl, 20 mM Tris-HCl (pH8.0), 1 mM EDTA, 0.5% NP-40 (v/v)).

### Cell-free protein expression and purification

Xpress-tagged proteins were synthesized using TNT-coupled *in vitro* transcription-translation system (cell-free rabbit reticulocyte expression system (Promega)) according to manufacturer’s instructions. TNT-coupled *in vitro* transcription-translated Xpress-tagged proteins were immunoprecipitated overnight at 4°C with an anti-Xpress antibody (Invitrogen) on protein A sepharose. Next day, the sepharose was washed three times with wash buffer (75 mM NaCl, 15 mM Tris-HCl, 1% bovine serum albumin).

### Co-immunoprecipitation (Co-IP)

Cells co-transfected with *Myc-ARX* and *V5-PICK1* or *HA-PKC* were lysed using extraction buffer 1 as described above and immunoprecipitated respectively with anti-Myc, anti-V5 (Invitrogen) or anti-HA antibodies overnight at 4°C under constant rotation. Protein A sepharose was added to immune complexes the next day for an hour at 4°C and then washed with Co-IP wash buffer (250 mM NaCl, 20 mM Tris-HCl (pH8.0), 1mM EDTA, 0.5% NP-40 (v/v)) three times. Immunoprecipitated proteins were eluted in 25μl pre-heated 60°C 1x SDS loading buffer (Invitrogen). Aliquots of protein supernatant were resolved on 4–12% SDS-PAGE gels and electrotransferred to nitrocellulose membranes to enable specific detection of precipitated proteins by immunoblotting. Membranes were blocked with 5% skim milk (w/v) in 1X TBS-T (10 mM Tris-base (pH 7.4), 150 mM NaCl, 0.05% Tween-20 and deionised water) for an hour at room temperature to block non-specific binding of secondary antibodies. The membranes were incubated with primary antibodies overnight at 4°C, washed in 1X TBS-T to remove excess antibody and then incubated with horseradish peroxidase (HRP) conjugated secondary antibodies for an hour at room temperature to detect antigen-antibody complexes. Following a final wash in 1X TBS-T, the protein signal was detected by enhanced chemiluminescence (ECL) (Amersham Pharmacia Biotech).

### Antibodies used for immunoblotting analyses

Primary antibodies include mouse anti-Myc antibody (1:1500) (Santa Cruz), mouse anti-HA antibody (1:15,000) (Sigma), mouse anti-Xpress antibody (1:1000) (Invitrogen), mouse HRP-conjugated anti-Myc antibody (1:5000),mouse HRP-conjugated anti-V5 antibody (1:5000) and rabbit anti-ARX antibody (1:2000) (In-house). Secondary antibodies include: goat anti-mouse HRP-conjugated antibody (1:1000) (Dako) and goat anti-rabbit Alexa 488 (1:2000) (Thermofisher). Antibodies used were diluted in either 1% or 3% milk (w/v) in TBS-T.

### In vitro kinase assay

Myc-tagged or Xpress-tagged ARX on protein A sepharose were washed twice with wash buffer and then twice with non-activated kinase buffer before the addition of activated kinase buffer. Different kinase buffers were prepared for enzyme specific kinase reactions as follows: kinase buffer A (100 μg/ml BSA and 100 μg/ml phosphatidlyserine were added into 40 mM Tris-HCl (pH7.4), 10 mM MgCl_2_, 0.4 mM CaCl_2_ to activate buffer A) for purified PKC (pool of PKC isozymes isolated from rat brain, Promega); kinase buffer B (100 μg/ml phosphatidlyserine and 20 μg/ml diacylglycerol were added into 40 mM Tris-HCl (pH7.4), 10 mM MgCl_2_, 0.4 mM CaCl_2_, 100 mM NaCl, 250 μM EGTA to activate buffer B) for recombinant PKC kinase, PRKCA and PRKCG (Sigma); kinase buffer C (1 mM NaF, 1 mM Na_3_VO_4_, 10 mM β-glycerolphosphate and 1 mM DTT were added into 50 mM HEPES (pH7.5), 10 mM MgCl_2_, 2.5 mM EGTA to activate buffer C) for constitutively active HA-tagged PKC isozymes. Precipitated proteins on sepharose were incubated with or without 25 ng purified PKC (Promega), 50 ng recombinant PKC (Sigma) or precipitated HA-PKC lysate in 30μl activated kinase buffer and 1–2 μCi of [γ-^32^P] ATP (PerkinElmer) at 30°C for 30 minutes. 500 μM of neurogranin peptide substrate (AAKIQASFRGHMARKK, Promega) or precipitated Xpress-tagged neurogranin protein, antibody and sepharose alone control, 100 μM of myristoylated PKC inhibitor peptide (myr-RFARKGALRQKNV, Promega) or 5 μM of PKC inhibitor GF109203X (Sigma) were included in the kinase assays. All reactions were terminated by the addition of concentrated 10X SDS loading buffer and boiling for 5 minutes. Protein samples were subjected to SDS-PAGE and autoradiography to detect phosphorylated proteins.

### Data analysis

All data are presented as mean ± SEM. Mean and SEM values were calculated from at least three independent experiments. The statistical significance of differences was measured by Student’s t-test and two-way ANOVA using Prism6. *P<0*.*05* was considered significant.

#### RNA sequencing analysis

RNA was isolated using Trizol (Invitrogen) and RNeasy Mini Kit (QIAGEN). For analysis, each group consisted of 4 technical replicates, collected across 4 different transfections. Samples were sequenced using Illumina HiSeq sequencing by AGTA Genomics, Adelaide, Australia obtaining 1x76bp reads with an average library size of 18.9 million reads per sample. The per base sequence quality was >95% bases above Q30 across all samples. Sequencing data was analysed on BaseSpace Sequencing Hub (Illumina Inc. 2016), with STAR aligner to Mus musculus/mm10 (RefSeq) with an average alignment of 95.6%. Aligned samples were analysed for differential expression using DESeq2 v1.1.0 through pairwise differential expression analysis between biological replicate groups with an average of 12,889 genes assessed after filtering. Data has been submitted to Gene Expression Omnibus under the GEO accession number: GSE105113. Validation of expression changes was done by quantitative RT-PCR (S7 Fig). Enrichment analysis was conducted in EnrichR [23] to check whether an input set of genes significantly overlaps with annotated gene sets. The significance of enrichment was selected based on the adjusted p-value using the Benjamini-Hochberg method for correction for multiple hypothesis testing. Genes with a log2 fold change with +/->1 cutoff value were used as the input into EnrichR [23]. GO Terms were ranked using the EnrichR method of combining the p-value computed using the Fisher exact test with the z-score of the deviation from the expected rank by multiplying these two numbers as follows: c = log(p) * z [23].

### Quantitative real-time polymerase chain reaction

For cDNA expression analysis, total RNA was isolated from transfected alpha-TC cells using Trizol (Invitrogen) and RNeasy Mini Kit (Qiagen). Routinely, 2μg of total RNA was primed with random hexanucleotides and then subjected to reverse transcription with Superscript III reverse transcriptase (Life technologies). Each group consisted of 3 technical replicates, collected across 3 different transfections. Validation of the reaction was done by PCR using primers specific to the ubiquitously expressed *B-Actin* (mouse). cDNAs were amplified using Taqman® Gene Expression Assay (Life Technologies) using the standard curve method for *Atf3* (Mm00476033_m1) and *Lgi3* (Mm00507490_m1). Probes of target genes were FAM-labelled. The housekeeping gene *Beta Actin* (Control mix; 4351315) was VIC-labelled and enabled normalisation of expression in each well. Standard curves for each target gene were prepared from alpha-TC cDNA at 10, 10^2^, 10^3^, 10^3^, and 10^4^ fold dilutions. All samples were run in triplicate. A typical TaqMan assay contained 1 x Fast Advanced Master Mix (Life Technologies), 0.75 μl control probe, 0.75 μl of test probe and 2 μl of cDNA in a final volume of 15 μl. Taqman® qPCR cycling conditions were an initial hold at 50°C for 2 mins, denaturation at 95°C for 20 sec, then 40 cycles of 95°C for 3 sec, and 60°C for 20 sec. Data analysis was done with StepOne™ Software (Life Technologies). Transcript levels are reported as relative difference to alpha-TC mock-transfected cells set at 1.0.

## Supporting information

S1 Table Enrichment analysis of biological processes of deregulated genes in alpha TC cells. Functional enrichment analysis of gene ontology (GO) terms for biological processes shows the differentially expressed genes due to ARX WT or ARX phosphorylation mutants compared to UT in alpha TC cells, with a log2 fold change with +/->1 cutoff value used as the input into EnrichR. The GO terms were ranked based on the combined EnrichR score of both the p-value (Fisher exact test) and the z-score (deviation from the expected rank) (Chen *et al* 2013, BMC Bioinformatics, 14, 128).
(XLSX)

S2 Table Enrichment analysis of cellular component of deregulated genes in alpha TC cells. Functional enrichment analysis of gene ontology (GO) terms for cellular component, shows the differentially expressed genes due to ARX WT or ARX phosphorylation mutants compared to UT in alpha TC cells, with a log2 fold change with +/->1 cutoff value used as the input into EnrichR. The GO terms were ranked based on the combined EnrichR score of both the p-value (Fisher exact test) and the z-score (deviation from the expected rank) (Chen *et al* 2013, BMC Bioinformatics, 14, 128).
(XLSX)

S3 Table Enrichment analysis of Molecular Function of deregulated genes in alpha TC cells. Functional enrichment analysis of gene ontology (GO) terms for molecular function, shows the differentially expressed genes due to ARX WT or ARX phosphorylation mutants compared to UT in alpha TC cells, with a log2 fold change with +/->1 cutoff value used as the input into EnrichR. The GO terms were ranked based on the combined EnrichR score of both the p-value (Fisher exact test) and the z-score (deviation from the expected rank) (Chen *et al* 2013, BMC Bioinformatics, 14, 128).
(XLSX)

S1 Fig Positive controls for anti-phospho antibodies. Treated HEK293T cells were included as positive controls; Pervanadate for pTry and CalyculinA for pThr.
(TIFF)

S2 Fig LC-ESI-IT-MS/MS identification of potential phosphorylation sites in ARX. LC-ESI-IT-MS/MS analysis of ARX-WT protein identified A, Serine 67 is phosphorylated (indicated by arrow) and is the only modifiable residue in phosphopeptide 2. B, in phosphopeptide 1 there were several potential residues that could be novel phosphorylation sites. Due to sufficient sequence coverage, the MS spectra can rule out serine 25, 26, 31 and tyrosine 27 as unlikely phosphorylation sites. Based on the spectra, the likely phosphorylation site of PP1 occurs either on serine 20, threonine 22 or serine 37. LC-ESI-IT-MS/MS analysis was performed as a fee for service by Adelaide Proteomics Centre, University of Adelaide, Australia.
(TIFF)

S3 Fig Schematic representation of 2DGE used to detect different protein isoforms of ARX. A) The first dimension is the separation of the proteins according to their isoelectric point. Second dimension is the electrophoretic separation of the proteins in the presence of sodium dodecyl sulphate (SDS) according to their molecular weights. Immunoblotting antibody-detection method was used to detect different isoforms of ARX proteins. B) 2DGE analyses of ARX-WT and ARX-S37A mutant. Total protein lysates of exogenously expressed full-length ARX-WT and ARX-S37A mutant proteins were subjected to isoelectric focusing on 24 cm pH 4.7–5.9 IPG strips. Proteins were then separated by SDS-PAGE and transferred to nitrocellulose membrane and immunoblotted with anti-Myc antibody. Immunoblot images are scanned and biostatistical analysis by software ‘R’ (performed by Adelaide Proteomics Centre, University of Adelaide, Australia) to determine difference states between ARX-WT and ARX-S37A mutant proteins.
(TIFF)

S4 Fig Protein Kinase C phosphorylates ARX *in vitro* when expressed in Hek293T cells, but not when expressed in a cell-free system. **A)** Myc-ARX exogenously expressed in Hek293T cells was immunoprecipitated with anti-Myc antibody and used as a substrate in a PKC *in vitro* kinase assay. Upon completion of the assay, reactions were terminated by addition of loading buffer and proteins were separated by SDS-PAGE. Presence of ARX protein was confirmed by immunoblotting (IB) with an anti-Myc antibody (right-hand panel). Myc-ARX (62 kDa) was phosphorylated by PKC (lane 2 top panel) as detected by autoradiography [^32^P]. When PKC inhibitor was added to the kinase reaction, the phosphorylation signal for ARX was abolished (lane 3 top panel). Mock-transfected HEK 293T protein lysate was included as both negative and background control (lane 1). A known PKC substrate, neurogranin (NGRN) was included in each assay as a positive control (lane 2 bottom panel). **B)** Repeat of (A) using cell-free expressed and precipitated Xpress-tagged proteins as substrates. Xpress-tagged ARX was not phosphorylated by PKC (lane 2 top panel). IP antibody and protein A sepharose complex was included in this assay as both negative and background control. These results are representative of at least two independent experiments.
(TIFF)

S5 Fig ARX interacts with PRKCA. HEK293T cells co-transfected with *Myc-ARX* and *HA-PRKCA* constructs were lysed and immunoprecipitated (IP) with antibodies against the Myc or HA tags. Precipitated proteins were separated on SDS-PAGE and analysed for the presence of co-immunoprecipitated proteins by immunoblotting (IB). In total protein lysate (bottom panel), HA-tagged *PRKCA* (75 kDa) was detected upon IB with anti-HA antibody while Myc-tagged ARX (62 kDa) was detected via IB with anti-Myc antibody. In the top panel, IP of co-transfected protein lysate with anti-HA and detection of bound Myc-tagged ARX protein by IB with anti-Myc antibody showed the interaction of ARX and PRKCA. Cells transfected with *Myc-ARX* alone or *HA-PRKCA* alone were also included as controls. A representative blot is shown from two independents experiments.
(TIFF)

S6 Fig Abolition of S37, S67 and S174 phosphorylation does not affect the transcriptional repression via homeodomain binding in a luciferase reporter assays nor sub-cellular localisation of ARX. **A)** Abolition of S37 and S67 phosphorylation does not affect the transcriptional activity of ARX using a luciferase reporter assay. ARX protein partner, co-repressor Groucho/TLE1 was added to determine the co-repression activity of ARX and TLE1 *in vitro*. **B**) Abolition of S174 by substitution with alanine does not affect the transcriptional activity of ARX in HEK293T cells in a luciferase reporter assay. Myc-tagged full-length ARX WT, phosphorylation mutant S174A, constitutively active S174E or Myc empty constructs were co-transfected with luciferase and Renilla (internal control) reporter constructs into HEK293T cells. Data were calculated as a ratio of luciferase to Renilla expression and expressed as a percentage of transcriptional activity relative to the activity of the Myc empty vector (100%). Error bars indicate SEM from three independent experiments.
**C)** ARX-WT and phosphorylation mutant proteins (ARX-S37A, ARX-S67A, ARX-S174A and ARX-S174E) show normal non-homogenous staining in the nucleus. The percentage of transfected cells displaying abnormal localisation was determined from ~200–300 transfected cells per construct from at least two separate transfection reactions, 24hr post-transfection. R332P mutation is an ARX mutant known to have abnormal localisation.
(TIFF)

S7 Fig Validation by quantitative RT-PCR of expression changes identified by RNASeq analysis. A selection of random genes was selected and tested by Taqman qRT-PCR in alpha TC cell in both untransfected and transfected with ARX-WT. Genes tested and probe ID is listed in S1 Text. The Log2FC calculated from qRT-PCR data was plotting against the RNASeq Log2FC data to show the level of correlation.
(TIFF)

S1 Text Experimental procedures and results. Supplementary experimental information is supplied regarding antibodies used, cloning of expression constructs, immunofluorescence and microscopy, 2D gel electrophoresis, isoelectric focusing and SDS-PAGE, Luciferase reporter assays and RT-qPCR validation of RNASeq data. Supplementary results are provided for Functional consequences of serine phosphorylation using a dual-luciferase reporter assay.
(DOCX)

## Abbreviations

2DGE: two-dimensional gel electrophoresis
ARX: Aristaless-related homeobox
CoIP: Co-immunoprecipitation
eh1: Engrailed homology repressor domain
HD: homeodomain
IB: immunoblot
IB: immunoblot
ID: intellectual disability
IP: immunoprecipitate
NGRN: neurogranin
OAR: aristaless domain
OP: octapeptide domain
PA: polyalanine tract
PICK1: Protein interacting with C kinase 1
PP: Phosphopeptides
PRKCA: protein kinase C alpha
TFBS: transcription factor binding site
