# Supplementary figures and images for: Regulating transcriptional activity by phosphorylation: A new mechanism for the ARX homeodomain transcription factor
Source: PLoS One. 2018 Nov 12;13(11):e0206914. doi: 10.1371/journal.pone.0206914 (PMC6231642; doi:10.1371/journal.pone.0206914)

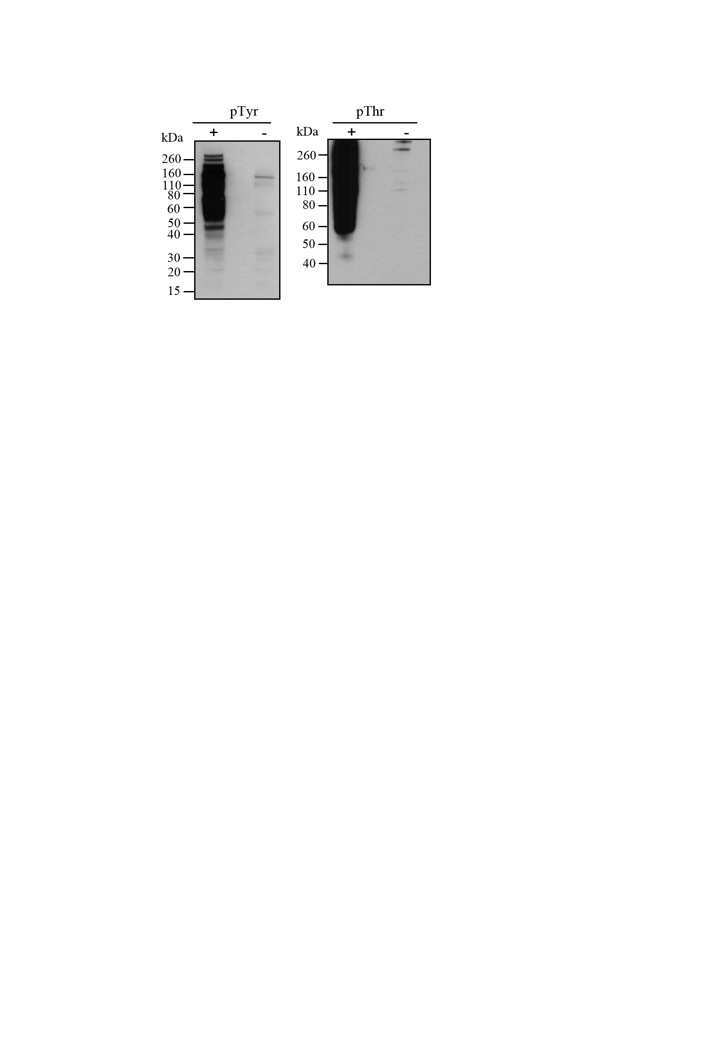

Supplement: S1 Fig — Treated HEK293T cells were included as positive controls; Pervanadate for pTry and CalyculinA for pThr. (TIFF) [file pone.0206914.s004.tiff]

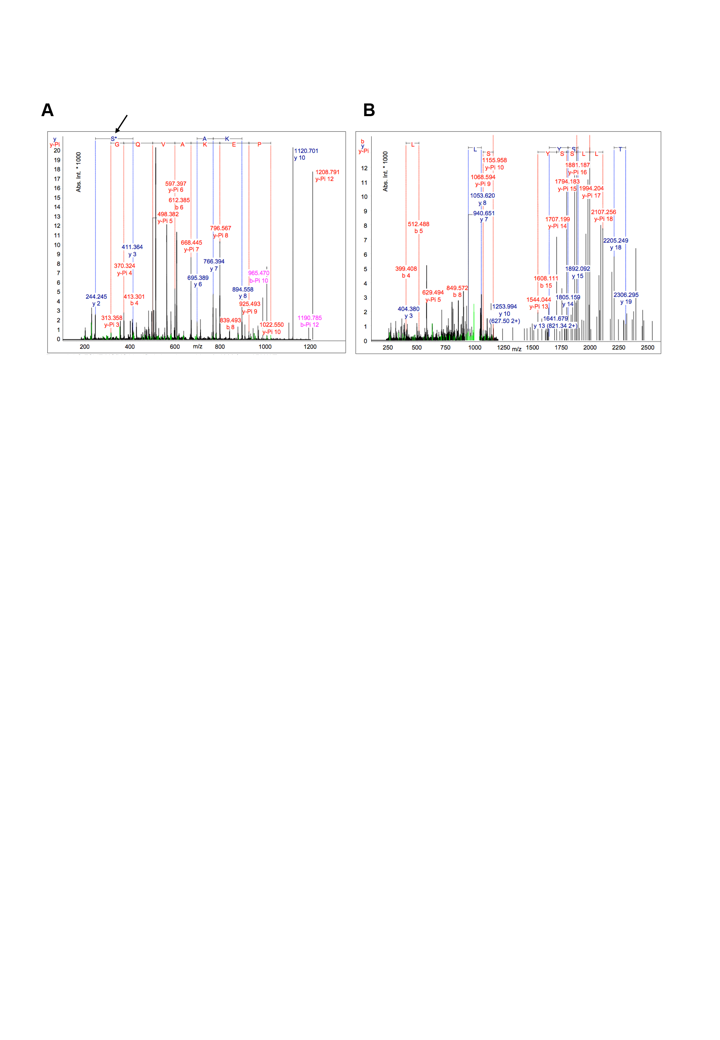

Supplement: S2 Fig — LC-ESI-IT-MS/MS analysis of ARX-WT protein identified A, Serine 67 is phosphorylated (indicated by arrow) and is the only modifiable residue in phosphopeptide 2. B, in phosphopeptide 1 there were several potential residues that could be novel phosphorylation sites. Due to sufficient sequence coverage, the MS spectra can rule out serine 25, 26, 31 and tyrosine 27 as unlikely phosphorylation sites. Based on the spectra, the likely phosphorylation site of PP1 occurs either on serine 20, threonine 22 or serine 37. LC-ESI-IT-MS/MS analysis was performed as a fee for service by Adelaide Proteomics Centre, University of Adelaide, Australia. (TIFF) [file pone.0206914.s005.tiff]

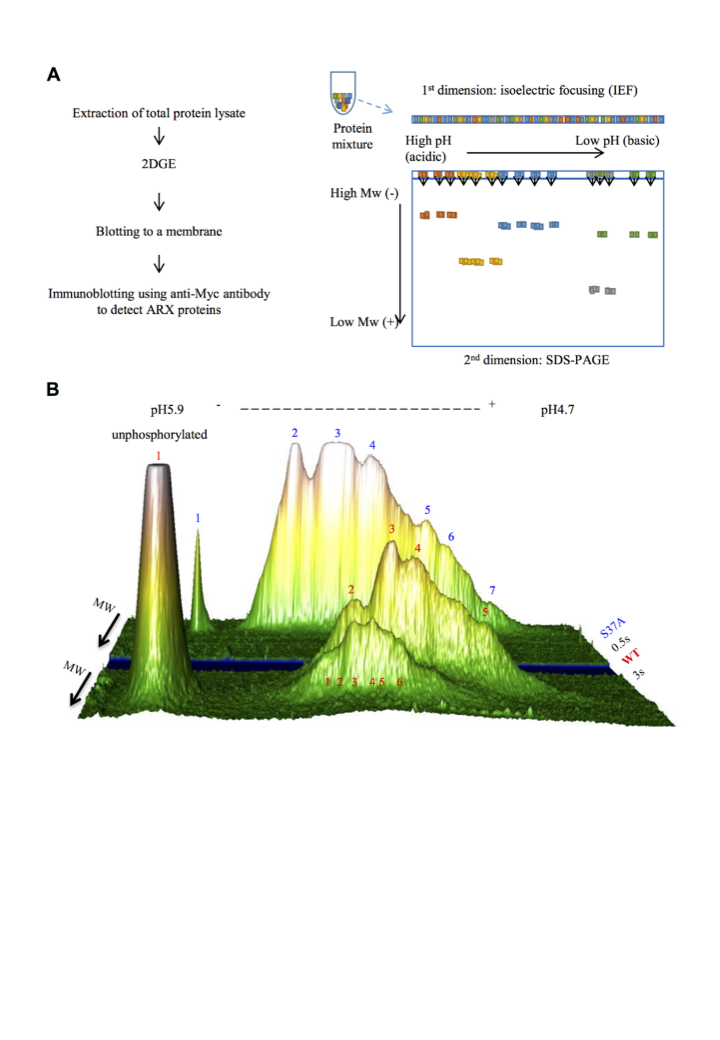

Supplement: S3 Fig — A) The first dimension is the separation of the proteins according to their isoelectric point. Second dimension is the electrophoretic separation of the proteins in the presence of sodium dodecyl sulphate (SDS) according to their molecular weights. Immunoblotting antibody-detection method was used to detect different isoforms of ARX proteins. B) 2DGE analyses of ARX-WT and ARX-S37A mutant. Total protein lysates of exogenously expressed full-length ARX-WT and ARX-S37A mutant proteins were subjected to isoelectric focusing on 24 cm pH 4.7–5.9 IPG strips. Proteins were then separated by SDS-PAGE and transferred to nitrocellulose membrane and immunoblotted with anti-Myc antibody. Immunoblot images are scanned and biostatistical analysis by software ‘R’ (performed by Adelaide Proteomics Centre, University of Adelaide, Australia) to determine difference states between ARX-WT and ARX-S37A mutant proteins. (TIFF) [file pone.0206914.s006.tiff]

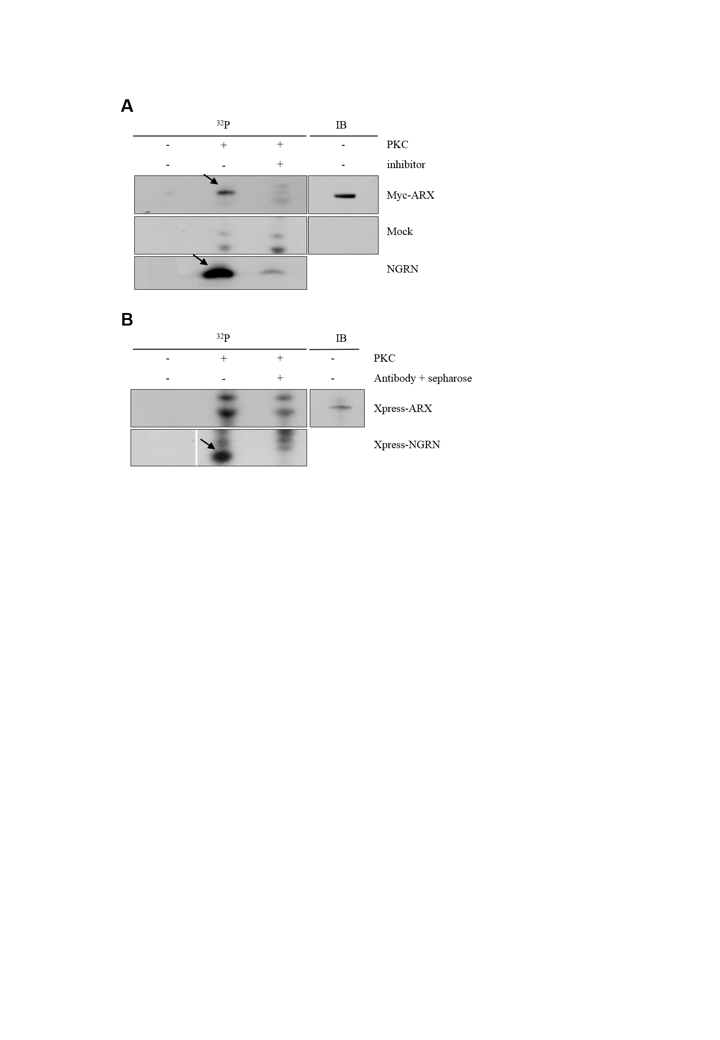

Supplement: S4 Fig — A) Myc-ARX exogenously expressed in Hek293T cells was immunoprecipitated with anti-Myc antibody and used as a substrate in a PKC in vitro kinase assay. Upon completion of the assay, reactions were terminated by addition of loading buffer and proteins were separated by SDS-PAGE. Presence of ARX protein was confirmed by immunoblotting (IB) with an anti-Myc antibody (right-hand panel). Myc-ARX (62 kDa) was phosphorylated by PKC (lane 2 top panel) as detected by autoradiography [32P]. When PKC inhibitor was added to the kinase reaction, the phosphorylation signal for ARX was abolished (lane 3 top panel). Mock-transfected HEK 293T protein lysate was included as both negative and background control (lane 1). A known PKC substrate, neurogranin (NGRN) was included in each assay as a positive control (lane 2 bottom panel). B) Repeat of (A) using cell-free expressed and precipitated Xpress-tagged proteins as substrates. Xpress-tagged ARX was not phosphorylated by PKC (lane 2 top panel). IP antibody and protein A sepharose complex was included in this assay as both negative and background control. These results are representative of at least two independent experiments. (TIFF) [file pone.0206914.s007.tiff]

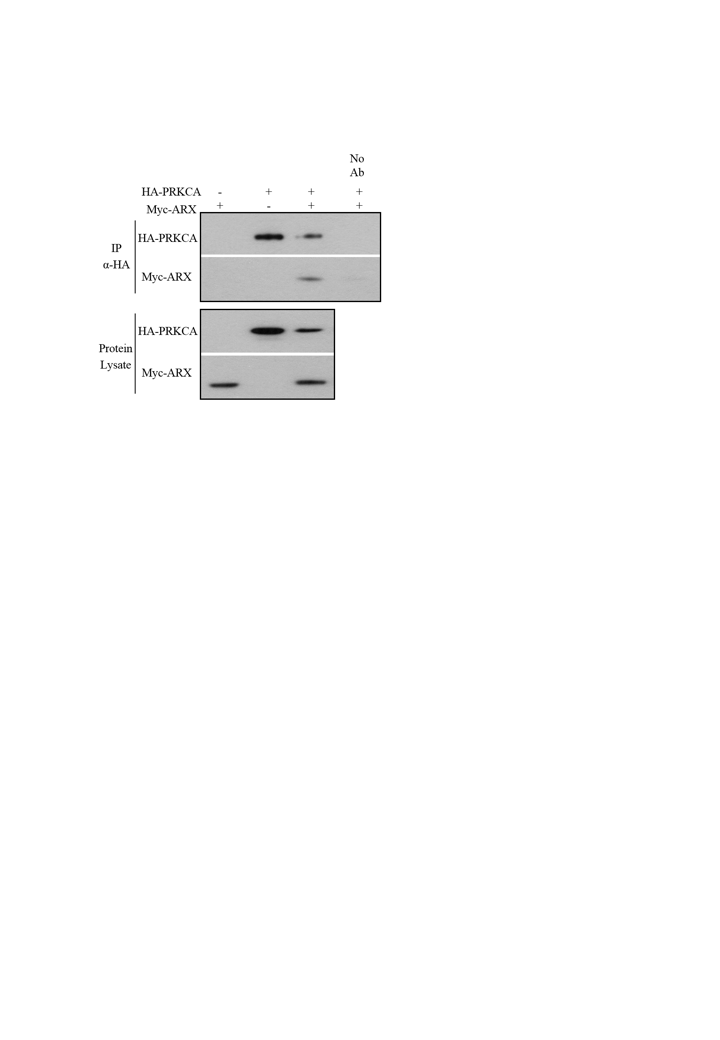

Supplement: S5 Fig — HEK293T cells co-transfected with Myc-ARX and HA-PRKCA constructs were lysed and immunoprecipitated (IP) with antibodies against the Myc or HA tags. Precipitated proteins were separated on SDS-PAGE and analysed for the presence of co-immunoprecipitated proteins by immunoblotting (IB). In total protein lysate (bottom panel), HA-tagged PRKCA (75 kDa) was detected upon IB with anti-HA antibody while Myc-tagged ARX (62 kDa) was detected via IB with anti-Myc antibody. In the top panel, IP of co-transfected protein lysate with anti-HA and detection of bound Myc-tagged ARX protein by IB with anti-Myc antibody showed the interaction of ARX and PRKCA. Cells transfected with Myc-ARX alone or HA-PRKCA alone were also included as controls. A representative blot is shown from two independents experiments. (TIFF) [file pone.0206914.s008.tiff]

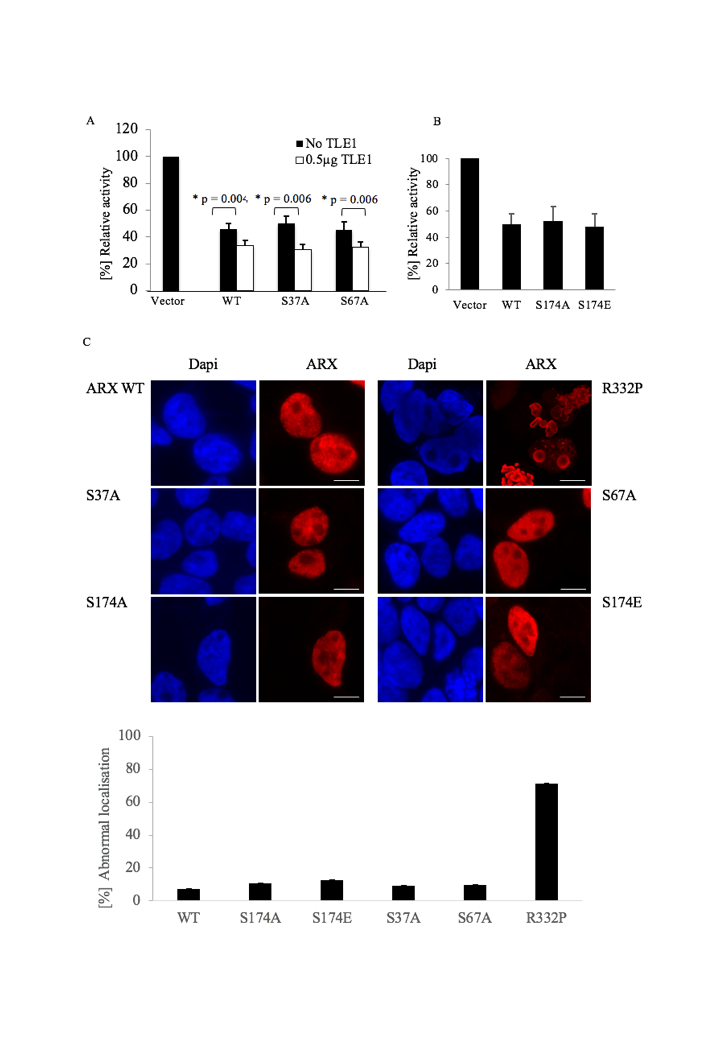

Supplement: S6 Fig — A) Abolition of S37 and S67 phosphorylation does not affect the transcriptional activity of ARX using a luciferase reporter assay. ARX protein partner, co-repressor Groucho/TLE1 was added to determine the co-repression activity of ARX and TLE1 in vitro. B) Abolition of S174 by substitution with alanine does not affect the transcriptional activity of ARX in HEK293T cells in a luciferase reporter assay. Myc-tagged full-length ARX WT, phosphorylation mutant S174A, constitutively active S174E or Myc empty constructs were co-transfected with luciferase and Renilla (internal control) reporter constructs into HEK293T cells. Data were calculated as a ratio of luciferase to Renilla expression and expressed as a percentage of transcriptional activity relative to the activity of the Myc empty vector (100%). Error bars indicate SEM from three independent experiments. C) ARX-WT and phosphorylation mutant proteins (ARX-S37A, ARX-S67A, ARX-S174A and ARX-S174E) show normal non-homogenous staining in the nucleus. The percentage of transfected cells displaying abnormal localisation was determined from ~200–300 transfected cells per construct from at least two separate transfection reactions, 24hr post-transfection. R332P mutation is an ARX mutant known to have abnormal localisation. (TIFF) [file pone.0206914.s009.tiff]

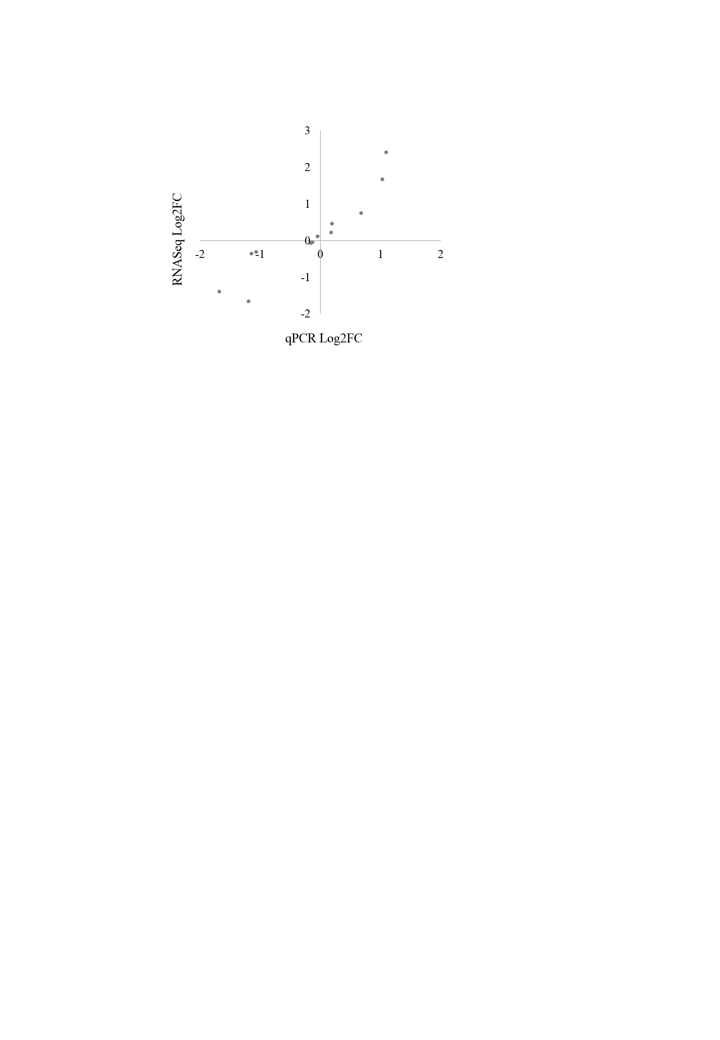

Supplement: S7 Fig — A selection of random genes was selected and tested by Taqman qRT-PCR in alpha TC cell in both untransfected and transfected with ARX-WT. Genes tested and probe ID is listed in S1 Text. The Log2FC calculated from qRT-PCR data was plotting against the RNASeq Log2FC data to show the level of correlation. (TIFF) [file pone.0206914.s010.tiff]
