# Supplementary material for: Regulating transcriptional activity by phosphorylation: A new mechanism for the ARX homeodomain transcription factor
Source: PLoS One. 2018 Nov 12;13(11):e0206914. doi: 10.1371/journal.pone.0206914 (PMC6231642; doi:10.1371/journal.pone.0206914)
Supplement: S1 Text — Supplementary experimental information is supplied regarding antibodies used, cloning of expression constructs, immunofluorescence and microscopy, 2D gel electrophoresis, isoelectric focusing and SDS-PAGE, Luciferase reporter assays and RT-qPCR validation of RNASeq data. Supplementary results are provided for Functional consequences of serine phosphorylation using a dual-luciferase reporter assay. (DOCX) [file pone.0206914.s011.docx]

**Supplementary data**

**Experimental procedures**

*Antibodies -* Primary antibodies used for immunofluorescence and/or immunoblotting: mouse anti-Myc antibody (1:1500) (Santa Cruz). The secondary antibodies were fluorescently labelled; goat anti-mouse-IgG conjugated to Cy3 (1:1000) (Jackson Laboratories, Maine, USA), goat anti-mouse-IgG Alexa Fluor 488 (1:2000) (Invitrogen). All antibodies used were diluted in 3% skim-milk (w/v) in TBS-T.

*Cloning of ARX expression constructs*

Glutamic acid (E) substitution was used to mimic phosphorylation (phosphomimetic mutant), with two changes required to achieve the substitution; c.520T>G & c.521C>A leading to p.S174E (ARX-S174E).

*Immunofluorescence and microscopy -* Transfected cells grown on sterile glass coverslips were harvested at 24 hours post-transfection by fixation in 3.7% formaldehyde-PBS. Fixed cells were permeabilised in 0.2% (v/v) Triton-PBS for 5 minutes and blocked with 5% skim-milk (w/v) in TBS-T for an hour at room temperature to avoid non-specific secondary antibody binding. After blocking, cells were incubated with primary antibody at 4°C overnight, followed by incubation with fluorescently labelled secondary antibody for an hour at room temperature. Removal of excess antibody was achieved with washes of TBS-T after each antibody incubation step. Nuclei were counterstained with DAPI (Molecular probes, Invitrogen) and the cells were examined by fluorescence microscopy. To determine the percentage of abnormal sub-cellular localisation in ARX transfected cells, ~200-300 transfected cells were counted for each construct from at least two different transient transfection experiments using a standard fluorescent microscope.

*2D-gel electrophoresis -* Urea-based protein extraction - Cell pellets harvested from *ARX* transfected cells were homogenised in 250 μl of IEF sample buffer TUC4 (7 M Urea, 2 M Thiourea, 30 mM Tris, 4% CHAPS, 1% Protease Inhibitor Pefabloc SC (Roche), 1% PSC protector reagent (Roche)) by pipetting. Cells were lysed using an ultrasonic probe for 30 pulses, cooled in ice-water for 2 minutes and sonicated again for 30 pulses. 10 μl of DNase I (Sigma-Aldrich) was added and the sample was passed through a syringe with 0.7mm tip for 20 times. The sample was incubated at room temperature for an hour and sonicated again as described above. Samples were centrifuged at *20,000 x g* at 15°C for 30 minutes. The supernatant collected was used for isoelectric focusing.

*Isoelectric focusing and SDS-PAGE - Sample clean-up prior to isoelectric focusing –* Cell pellets harvested from ARX transfected cells were homogenised in 250 μl of IEF sample buffer TUC4 (7 M Urea, 2 M Thiourea, 30 mM Tris, 4% CHAPS, 1% Protease Inhibitor Pefabloc SC (Roche), 1% PSC protector reagent (Roche)) by pipetting. Cells were lysed using an ultrasonic probe for 30 pulses, cooled in ice-water for 2 minutes and sonicated again for 30 pulses. Addition of 10 μl of DNase I (Sigma-Aldrich) to the sample was passed through a syringe with 0.7mm tip for 20 times. The sample was incubated at RT for an hour and sonicated again as described above. Afterwards, the samples were centrifuged at *20,000 x g* at 15°C for 30 minutes. The supernatant collected into 1.5 ml microcentrifuge tubes and stored at -80°C until further use.

IPG strips (24cm) with pH range from 4.2-5.9 were rehydrated overnight in rehydration buffer containing 6 M urea, 2 M thiourea, 1% CHAPS, 0.5% 3-11 NL carrier ampholytes and 200 mM 2,2 Dithiodiethanol (HED) (GE Healthcare). 50 μg of protein of each sample was applied to the IPG strips by cup-loading. Focusing was performed at 8000V for 90,000 Vhours with the current limited to 50 μA per strip. The second dimension SDS-PAGE was carried out on home casted 18 x 25 cm^2^ polyacrylamide gels of 12.5%. The equilibrated strips were laid onto the gels and sealed into place with agarose (1% LMP agarose in electrophoresis buffer (192mM Glycine, 50mM Tris, 0.1%SDS)). SDS-PAGE was performed at 5 mA/gel for an hour, 8 mA/gel for an hour and 20 mA/gel until the bromophenol blue front had reached the end of the gel. After SDS-PAGE the proteins were electroblotted at room temperature onto a nitrocellulose membrane using a semi-dry transfer system (GE Healthcare). The transfer buffer consisted of 25 mM Tris, 192 mM Glycine and 0.1% SDS (w/v) in 10% Methanol. The nitrocellulose membrane was wetted in 20% Methanol for 3 minutes before use. The proteins were transferred by applying a tension of 30 V for an hour. Western immunoblot was performed as described before. After detection of the target protein by antibodies and ECL, the membrane was scanned (Image Scanner, GE Healthcare) and visualised by the software “R”.

*Luciferase reporter assay - Myc* empty vector or *Myc-ARX* Wild-type or phospho-mutants were co-transfected with *pGL4.13 [luc2/SV40]* firefly luciferase reporter plasmid (Promega) and a *pGL4.74 [hRluc/TK]* Renilla reporter plasmid (internal control for transcription efficiency) (Promega) into HEK293T cells using Lipofectamine 2000 (Invitrogen). Firefly and Renilla luciferase activities were assayed 24 hours post-transfection, using dual-luciferase reporter assay system (Promega) according to the manufacturer’s protocol. In three independent transfections, each test sample was carried out in replicate, with triplicates of each replicate measured in the reporter assay. The firefly luciferase activity was normalized according to the corresponding *Renilla* luciferase activity in each well, and the ratio of luciferase activity was reported relative to pCMV-Myc empty vector within each transfection. Results are expressed as a percentage of transcriptional activity relative to the activity of the Myc empty vector (100%).

*RT-qPCR – RNASeq Validation -* RNASeq results were validated using Taqman RT-qPCR on alpha TC either untransfected or transfected with ARX-WT. RNA was extracted using Trizol (Thermofisher) and RNeasy Mini Kit (Qiagen) and treated with DNase 1 (Qiagen) according to the manufacturer’s instruction. cDNA was prepared as described in SuperScript II reverse transcriptase (Thermofisher) manual with 1 μg of RNA primed by random hexanucleotides. Along with samples, template negative and reverse transcriptase negative controls were included to determine product specificity. Genes selected for validation studies were assayed as described in Taqman^®^ PreAmp Master Mix Kit user guide (Applied Biosystem). For each validation gene quantified with a Taqman probe labelled with FAM, the expression values were normalized to the reference gene Gapdh assayed in the same well using the TaqMan probe labelled with VIC. TaqMan probes used in this study are: Aff1 (MM00836035_M1), Atf3 (MM00476033_M1), Calr (MM00482936_M1), Chga (MM00514341_M1), Ddost (MM00492100_M1), GAPDH (MM99999915_G1), Gcg (MM00801714_M1), Gnb1 (MM00515002_M1), Hey1 (MM00468865_M1), Jun (MM00495062_S1), Pcbd2 (MM01342270_M1), Rhob (MM00455902_S1), Timm8a1 (MM02602707_G1),

**Supplementary results**

***Functional consequences of serine phosphorylation using a dual-luciferase reporter assay*** *-* We have previously shown that the transcriptional repression activity of ARX at the N-terminus is mediated in part by its interaction with the co-repressor Groucho/TLE1, via binding to the octapeptide domain (1). Given the N-terminal residues in question, serine 37 in particular, are in close proximity to the Groucho/TLE1 binding region (Figure 2D, OP underlined) we set out to measure the impact of phosphorylation on ARX transcriptional activity. The luciferase reporter vector (containing was co-transfected with Myc-vector or Myc-ARX alone into HE293T cells and the luciferase activity in these transfected cells was measured 24 hours post-transfection. Prior to use in luciferase assays, all plasmids were confirmed to yield consistent transfection efficiencies (manual counting of immunofluorescent cells) and equivalent amounts of expressed protein (immunoblotting) across test concentrations of plasmid DNA transfected into cells for luciferase assay analysis. Within the luciferase reporter assay, the co-transfection of a Renilla reporter vector controls for transfection efficiency. The expression of luciferase was normalised to Renilla expression in each sample. To compare across replicate experiments, the ratio of luciferase to Renilla expression for the Myc-vector was set to 100% in all analyses. Compared to the Myc-vector control, the relative activity of Myc-ARX in this assay is reduced to 43% and is consistent with transcriptional repression observed in previous studies (2). When the phosphorylation null mutants were tested there was no significant difference in their relative activity compared to ARX-WT (Supplementary Figure 6a, black bars) indicating that the lack of phosphorylation at serine 37 or 67 does not interfere with ARX activity via binding to the 3xTFBS when measured in HEK293T cells. Addition of Groucho/TLE1 to the luciferase reporter assay displayed the expected reduction in relative activity compared to ARX-WT alone, indicating an increased repression with the addition of the co-repressor (Supplementary Figure 6a, white bars). A similar trend was noted for both ARX phosphorylation null mutants, suggesting that the abolition of phosphorylation at serine 37 or 67 did not have any measurable impact on the transcriptional co-repression activity of ARX by Groucho/TLE1 in this reporter assay, at least in HEK293T cells. To examine if PKC mediated p.Ser174 phosphorylation regulates the transcriptional activity of ARX, phospho-null (p.S174A) and phosphomimetic (p.S174E) mutants were created in full-length ARX. Myc-ARX wildtype and phosphorylation mutant constructs were co-transfected with luciferase reporters into HEK293T cells. Similar to ARX-WT, both phosphorylation mutant Myc-tagged ARX proteins repressed the luciferase activity by ~50% (Supplementary Figure 6b, black bars), indicating that phosphorylation of serine 174 alone has no significant effect on the repression activity of ARX using a luciferase reporter assay in HEK293T cells. Abolition of serine 37, 67 or 174 phosphorylation did not affect nuclear localisation of ARX which was similar to that of ARX-WT in comparison to the mislocalisation of the R333P mutation in ARX (Supplementary Figure S6c), which also displays a loss of transcriptional activity (2).

1. McKenzie, O., Ponte, I., Mangelsdorf, M., Finnis, M., Colasante, G., Shoubridge, C., Stifani, S., Gecz, J. and Broccoli, V. (2007) Aristaless-related homeobox gene, the gene responsible for West syndrome and related disorders, is a Groucho/transducin-like enhancer of split dependent transcriptional repressor. *Neuroscience*, 146, 236-247.
2. Shoubridge, C., Tan, M.H., Seiboth, G. and Gecz, J. (2012) ARX homeodomain mutations abolish DNA binding and lead to a loss of transcriptional repression. *Hum Mol Genet*, 21, 1639-1647.
